# Whole blood transcriptomics reveals sepsis mortality-associated changes in neutrophil degranulation

**DOI:** 10.1093/ajrcmb/aanag021

**Published:** 2026-02-21

**Authors:** Heather M Giannini, Mengyuan Kan, Christopher V Cosgriff, Michael P Morley, Todd A Miano, Nisha Narayanan, Caroline A G Ittner, Alexandra P Turner, Mika P Esperanza, Matthew C Erlich, Oluwatosin Oniyide, Brian J Anderson, Tiffanie K Jones, Rui Feng, John P Reilly, Blanca E Himes, Michael G S Shashaty, Nuala J Meyer

**Affiliations:** Division of Pulmonary, Allergy, and Critical Care Medicine, Department of Medicine, University of Pennsylvania Perelman School of Medicine, Philadelphia, PA, United States; Center for Translational Lung Biology, Lung Biology Institute, University of Pennsylvania Perelman School of Medicine, Philadelphia, PA, United States; Department of Epidemiology, Biostatistics and Informatics, University of Pennsylvania Perelman School of Medicine, Philadelphia, PA, United States; Division of Pulmonary and Critical Care Medicine, Department of Medicine, Massachusetts General Hospital, Boston, MA, United States; Center for Translational Lung Biology, Lung Biology Institute, University of Pennsylvania Perelman School of Medicine, Philadelphia, PA, United States; Department of Medicine, University of Pennsylvania Perelman School of Medicine, Philadelphia, PA, United States; Center for Clinical Epidemiology and Biostatistics, University of Pennsylvania Perelman School of Medicine, Philadelphia, PA, United States; Department of Epidemiology, Biostatistics and Informatics, University of Pennsylvania Perelman School of Medicine, Philadelphia, PA, United States; Division of Pulmonary, Allergy, and Critical Care Medicine, Department of Medicine, University of Pennsylvania Perelman School of Medicine, Philadelphia, PA, United States; Center for Translational Lung Biology, Lung Biology Institute, University of Pennsylvania Perelman School of Medicine, Philadelphia, PA, United States; Division of Pulmonary, Allergy, and Critical Care Medicine, Department of Medicine, University of Pennsylvania Perelman School of Medicine, Philadelphia, PA, United States; Center for Translational Lung Biology, Lung Biology Institute, University of Pennsylvania Perelman School of Medicine, Philadelphia, PA, United States; Division of Pulmonary, Allergy, and Critical Care Medicine, Department of Medicine, University of Pennsylvania Perelman School of Medicine, Philadelphia, PA, United States; Center for Translational Lung Biology, Lung Biology Institute, University of Pennsylvania Perelman School of Medicine, Philadelphia, PA, United States; Division of Pulmonary, Allergy, and Critical Care Medicine, Department of Medicine, University of Pennsylvania Perelman School of Medicine, Philadelphia, PA, United States; Center for Translational Lung Biology, Lung Biology Institute, University of Pennsylvania Perelman School of Medicine, Philadelphia, PA, United States; Division of Pulmonary, Allergy, and Critical Care Medicine, Department of Medicine, University of Pennsylvania Perelman School of Medicine, Philadelphia, PA, United States; Center for Translational Lung Biology, Lung Biology Institute, University of Pennsylvania Perelman School of Medicine, Philadelphia, PA, United States; Division of Pulmonary, Allergy, and Critical Care Medicine, Department of Medicine, University of Pennsylvania Perelman School of Medicine, Philadelphia, PA, United States; Center for Translational Lung Biology, Lung Biology Institute, University of Pennsylvania Perelman School of Medicine, Philadelphia, PA, United States; Division of Pulmonary, Allergy, and Critical Care Medicine, Department of Medicine, University of Pennsylvania Perelman School of Medicine, Philadelphia, PA, United States; Center for Translational Lung Biology, Lung Biology Institute, University of Pennsylvania Perelman School of Medicine, Philadelphia, PA, United States; Department of Epidemiology, Biostatistics and Informatics, University of Pennsylvania Perelman School of Medicine, Philadelphia, PA, United States; Department of Epidemiology, Biostatistics and Informatics, University of Pennsylvania Perelman School of Medicine, Philadelphia, PA, United States; Division of Pulmonary, Allergy, and Critical Care Medicine, Department of Medicine, University of Pennsylvania Perelman School of Medicine, Philadelphia, PA, United States; Center for Translational Lung Biology, Lung Biology Institute, University of Pennsylvania Perelman School of Medicine, Philadelphia, PA, United States; National Heart, Lung, and Blood Institute, National Institutes of Health, Bethesda, MD, United States; Division of Pulmonary, Allergy, and Critical Care Medicine, Department of Medicine, University of Pennsylvania Perelman School of Medicine, Philadelphia, PA, United States; Center for Translational Lung Biology, Lung Biology Institute, University of Pennsylvania Perelman School of Medicine, Philadelphia, PA, United States; Center for Clinical Epidemiology and Biostatistics, University of Pennsylvania Perelman School of Medicine, Philadelphia, PA, United States; Division of Pulmonary, Allergy, and Critical Care Medicine, Department of Medicine, University of Pennsylvania Perelman School of Medicine, Philadelphia, PA, United States; Center for Translational Lung Biology, Lung Biology Institute, University of Pennsylvania Perelman School of Medicine, Philadelphia, PA, United States

**Keywords:** critical illness, neutrophil degranulation, sepsis mortality, whole blood transcriptome

## Abstract

Transcriptomic analysis of blood cells can reveal key elements of the dysregulated host response in sepsis and spur biomarker and mechanism identification. We hypothesized that sepsis nonsurvivors exhibit a distinct transcriptional signature in whole blood that reflects insights into sepsis mortality. We conducted a prospective observational cohort study of 161 critically ill sepsis patients. Whole blood RNA was collected within 24 hours of intensive care unit admission. Gene expression levels were measured using microarrays, and changes in gene levels were compared between 30-day nonsurvivors and survivors, adjusting for age, sex, and neutrophil count. Pathway overrepresentation analysis and weighted gene co-expression analysis were performed to identify biological pathways and gene co-expression groups, respectively, associated with sepsis mortality. Gene- and pathway-based results were compared to findings in an independent cohort of 479 sepsis patients with 28-day mortality data. Thirty-day mortality in the enrolled sepsis cohort was 37% (60 of 161 patients). We identified 1106 differentially expressed genes in nonsurvivors (Benjamini-Hochberg-adjusted *P*-value <.05), including several neutrophil-related genes (*CEACAM8*, *ELANE*, *PRTN3*, *MPO*, *CEACAM6*, *DEFA4*, *MS4A3*) with expression levels over 1.8 times higher in nonsurvivors despite adjusting for neutrophil counts. The neutrophil degranulation pathway was prominent based on its overrepresentation in (1) differentially expressed genes in both cohorts, (2) overrepresentation by gene set enrichment analysis, and (3) 4 of the 6 gene co-expression groups correlated with sepsis mortality. Our findings highlight the involvement of neutrophil degranulation genes in sepsis mortality, prompting further study to better understand whether they constitute a modifiable target.

## Introduction

Sepsis is the leading cause of death in U.S. hospitals and affects nearly 50 million people globally per year.^1^ Sepsis is defined as the dysregulated host response to infection,^2^ however, identifying specific and targetable immune abnormalities for therapeutic ­intervention has been challenging. An effective immune response clears pathogens but also protects host tissues from collateral damage. In sepsis, broad immune dysregulation causes organ failure and increased risk of mortality. Therapeutic trials targeting known inflammatory markers in sepsis have failed to improve ­outcomes.^3^ Efforts to identify sepsis therapies may have been thwarted by a focus on the wrong targets, undetected or unaccounted for heterogeneity among study participants, or issues of timing in a highly dynamic immune response and clinical syndrome.^4^

Whole blood gene expression has the potential to identify unique components of the host immune response that contribute to sepsis pathogenesis. There are well-defined transcriptional changes that distinguish a quiescent immune system from sterile inflammation and the profound dysregulation of sepsis,^5^^,^^6^ but these differences have been difficult to distill into clinically useful information. Furthermore, gene expression changes associated with sepsis mortality are not well understood. Whole blood represents an easily accessible and frequently sampled tissue compartment that has potential for the development of transcriptome-based diagnostic,^7-9^ prognostic,^10^ and predictive biomarkers.^11^^,^^12^ Peripheral blood dynamics parallel the clinical trajectory and can be developed for point-of-care testing. Application of unsupervised machine learning to whole blood gene expression data has identified subgroups of sepsis patients based on characteristics that are not clinically evident yet have distinct immune patterns, some of which are associated with sepsis mortality.^13-15^ The results of these studies suggest that the identification of mortality-dysregulated transcripts may reveal unexpected sepsis pathology or prompt development of novel targets for immune-focused sepsis interventions. Although publicly available transcriptomic data have been widely used to predict patients at high risk of sepsis mortality,^10^^,^^15^ studies comparing gene expression between sepsis survivors and nonsurvivors remain limited, hindering independent replication of mortality-associated expression signatures.

Here, we obtained gene expression data corresponding to admission to an intensive care unit (ICU) (ie, within 24 hours of ICU admission) to identify gene expression signatures of sepsis mortality and associated biological pathways. We further leveraged transcriptomic data from a large independent cohort of participants with sepsis mortality information to attempt to replicate our findings.

## Materials and methods

Additional methods are detailed in the online supplement.

### Study population

This study was approved by the University of Pennsylvania Institutional Review Board. Patients were enrolled in the Molecular Epidemiology of SepsiS in the ICU (MESSI) cohort as previously described.^16^  Figure S1 depicts the enrollment of 161 participants for the gene expression study. Mortality was defined as death occurring within 30 days after ICU admission.

### Gene expression microarray and data analysis

Total RNA from 161 whole blood samples was isolated and underwent transcriptomic profiling on Affymetrix Human Genome ST 2.1 array. Raw intensity files were analyzed using the RAVED pipeline (https://github.com/HimesGroup/raved).^17^ Pairwise comparison assessed expression differences in nonsurvivors vs survivors, adjusting for age, sex, and neutrophil count using the R package *limma* (v3.34.9).^18^  *P*-values were adjusted for multiple correction using Benjamini-Hochberg (BH) procedure. The transcriptomic data are deposited in the Gene Expression Omnibus (GEO) under accession number GSE272769. We report all differentially expressed genes (DEGs) achieving the BH-adjusted *P*-value <.05 and focus on those with absolute log_2_ fold change (FC) ≥0.85 (ie, FC ≥1.80 or ≤0.56) to balance parsimony and potential biological significance. To account for potential influence of infection or immunosuppression on mortality-associated genes, we repeated the comparison of survivors and nonsurvivors adjusting for bacteremia or immunocompromised status.

### Replication cohort

To test whether our results generalized to an independent cohort, we analyzed transcriptomic data from the Molecular Diagnosis and Risk Stratification of Sepsis (MARS) project (GSE65682).^14^ Patient samples were collected within the first 24 hours of ICU admission, with survival monitored for 28 days. Transcriptomic data of 479 subjects with sepsis mortality information were analyzed using the same RAVED pipeline.^17^ Pairwise comparison assessed expression differences in sepsis nonsurvivors vs survivors, adjusting for age and sex. We report a trans-cohort (MESSI + MARS) gene set of overlapping DEGs (BH-adjusted *P*-value <.05) that exhibit consistent direction of expression changes in nonsurvivors.

### Neutrophil-specific gene expression levels

To understand cell specificity, neutrophil gene expression levels of select genes were retrieved from the Immune Cell Gene Expression Atlas from the University of Tokyo (ImmuNexUT) (https://www.immunexut.org/).^19^

### Hierarchical clustering

Hierarchical clustering was performed to cluster DEGs meeting BH-adjusted *P*-value <.05 in MESSI, including (1) the parsimonious set limited to those with absolute log_2_FC ≥ 0.85 and (2) DEGs with absolute log_2_FC ≥ 0.5, as well as (3) the trans-cohort (MESSI + MARS) set of concordant DEG.

### Predictive model

Predictive models for sepsis mortality were developed from the MESSI parsimonious and trans-cohort gene sets described above using LASSO logistic regression implemented in the R package *glmnet*. Model performance was evaluated using the Area Under the Receiver Operating Characteristic curve (AUROC).

### Pathway overrepresentation analysis

Pathway overrepresentation analysis was performed with KEGG and Reactome pathway annotations using 2 approaches: (1) *functional enrichment analysis*, using a modified Fisher’s exact test,^20^ which assessed whether genes from a specific pathway were overrepresented in a gene set of interest compared to their occurrence in the background genome; and (2) *gene-set enrichment analysis* (*GSEA*)^21^ which identified pathways overrepresented in a ranked gene list, ie, all the genes previously tested ordered by their differential expression results. GSEA determines key genes driving this overrepresentation and the overall directionality of these pathway genes’ influence on sepsis mortality.

### Weighted gene co-expression network analysis

Weighted gene co-expression network analysis (WGCNA) was conducted using the WGCNA R package^22^ to identify groups of genes with similar expression patterns and assess their correlation with phenotypic characteristics.

### Cytokine analysis

We measured five day-0 cytokines (interleukin (IL)-6, IL-8, IL-10, IL-1β, and IL-1 receptor antagonist (IL-1RA)) in 151 MESSI participants (94%) from the gene expression subcohort using electrochemiluminescence (MesoScale Discovery, Rockville, MD) as described.^23^ Cytokine concentrations were log-transformed for normality and used to test for correlation with gene co-expression groups identified by WGCNA.

## Results

Demographic and clinical characteristics of the 1773 patients enrolled in the MESSI cohort and of the 161 participants in the MESSI gene expression subcohort are depicted in Table S1 and Table 1, respectively. The gene expression subcohort was largely representative of the parent cohort with the exception of a higher proportion of participants requiring vasopressors (*P*-value <.001) and slight differences in the proportion with immunocompromise and pulmonary or gastrointestinal primary source of sepsis. Among the 161 patients in the subcohort, 60 (37%) did not survive 30 days. Compared to survivors, sepsis nonsurvivors were older (*P*-value = .01) and had higher acute physiology and chronic health evaluation (APACHE) III scores (*P*-value <.001), higher prevalence of chronic dialysis (*P* = .006), higher measured lactate (*P*-value <.001), and a higher proportion with immune suppression (*P*-value = .07). Most patients demonstrated septic shock physiology (123 of 161 total patients, 76%), requiring administration of vasopressor at the time of enrollment. The proportion of patients with septic shock was higher in nonsurvivors with nominal significance (*P*-value = .06). The MARS cohort was comprised of 114 nonsurvivors and 365 survivors within 28 days after ICU admission, with a 28-day sepsis mortality rate of 24% (Table 2). Consistent with MESSI trends, MARS nonsurvivors were older (*P*-value = .01) and had more frequent ICU-acquired infection, with nominal significance (*P*-value = .09). Most other characteristics available in the MESSI cohort were not available in GEO for the MARS cohort, precluding other comparisons.

**Table 1:** Demographic and clinical characteristics of the 161 MESSI gene expression participants.

Characteristics	Overall (*N* = 161)	30-day nonsurvivors (*N* = 60)	30-day survivors (*N* = 101)	*P*-value
**Age (years)**	63 (54, 73)	66 (58, 75)	61 (51, 70)	.01
**Male**	74 (46%)	30 (50%)	44 (44%)	.51
**Race/ethnicity**				
** White**	100 (62%)	38 (63%)	62 (61%)	.37
** Black**	53 (33%)	18 (30%)	35 (35%)	
** Asian**	6 (4%)	4 (7%)	2 (2%)	
** Others**	2 (1%)	0 (0%)	2 (2%)	
** Hispanic**	7 (4%)	1 (2%)	6 (6%)	.26
**APACHE III score**	92 (66, 125)	121 (92, 147)	75 (60, 107)	<.001
**Medical comorbidities**				
** Immune suppression**	74 (46%)	33 (56%)	41 (41%)	.07
** Hematologic malignancy**	19 (12%)	8 (13%)	11 (11%)	.63
** Solid malignancy**	47 (29%)	25 (42%)	22 (22%)	.01
** Diabetes mellitus**	55 (34%)	19 (32%)	36 (36%)	.73
** Chronic dialysis**	24 (15%)	15 (25%)	9 (8.9%)	.006
**Sepsis features**				
** Septic shock**	123 (76%)	51 (85%)	72 (71%)	.06
** Culture positive^a^**	115 (71%)	48 (80%)	67 (66%)	.07
** Bacteremia**	69 (43%)	31 (52%)	38 (38%)	.09
** Gram-negative source**	66 (41%)	29 (48%)	37 (37%)	.15
** Gram-positive source**	63 (39%)	25 (42%)	38 (38%)	.61
** Viral source**	12 (7%)	3 (5%)	9 (9%)	.37
** Fungal source**	10 (6%)	5 (8%)	5 (5%)	.40
**Primary source**				
** Pulmonary**	60 (37%)	20 (33%)	40 (40%)	.37
** Gastrointestinal**	34 (21%)	17 (28%)	17 (17%)	
** Genitourinary**	21 (13%)	5 (8%)	16 (16%)	
** Bloodstream**	19 (12%)	9 (15%)	10 (10%)	
** Other**	13 (8%)	4 (7%)	9 (9%)	
** Unclear**	14 (9%)	5 (8%)	9 (9%)	
**Lactate (mmol/L)**	1.99 (1.35, 3.49)	2.85 (1.54, 5.62)	1.93 (1.29, 3.07)	<.001
**White blood cell count (10^9^ cells/L)**	13 (8, 18)	14 (9, 20)	12 (7, 17)	.20
**Neutrophil count (10^9^ cells/L)**	11 (6, 16)	11 (6, 18)	11 (6, 15)	.42
**Lymphocyte count (10^9^ cells/L)**	0.60 (0.30, 1.02)	0.68 (0.30, 1.13)	0.51 (0.30, 1.00)	.52
**Neutrophil-to-lymphocyte ratio (NLR)**	18 (8, 39)	19 (8, 38)	18 (9, 40)	.98

Continuous variables are shown as median (1 quartile, 3 quartile). Categorical variables are shown as *n* (%). *P*-values were calculated with Fisher’s exact test for categorical variables and the Wilcoxon rank-sum test for continuous variables.

a Culture positive includes any microbiologically confirmed infection between day −7 to day +7 centered around ICU admission. All culture and molecular tests during this window were reviewed. 28% (36/161) of participants had more than 1 positive result; 17% (28/161) of participants had both Gram-positive and Gram-negative organisms identified, and 58% (7/12) of participants with a viral infection also exhibited a bacterial infection at enrollment.

Abbreviation: APACHE, acute physiology and chronic health evaluation.

**Table 2:** Demographic and clinical characteristics of the MARS participants.

The MARS cohort				
Characteristics	Overall (*N* = 479)	28-day nonsurvivors (*N* = 114)	28-day survivors (*N* = 365)	*P*-value
**Age (years)**	63 (53, 71)	67 (56, 74)	63 (50, 71)	.01
**Male**	272 (57%)	68 (59.6%)	204 (55.9%)	.52
**Medical comorbidities**				
** Diabetes mellitus**	89 (23%)	22 (24%)	67 (22%)	.67
**Pneumonia diagnoses**				
** Community-acquired pneumonia**	106 (58%)	23 (57.5%)	83 (58%)	1.00
** Hospital-acquired pneumonia**	77 (42%)	17 (42.5%)	60 (42%)	
**ICU-acquired infection**	46 (14%)	15 (20.8%)	31 (12.4%)	.09
**Thrombocytopenia**				
** Very low**	17 (17.9%)	6 (21.4%)	11 (16.4%)	.83
** Medium low**	30 (31.6%)	10 (35.7%)	20 (29.9%)	
** Low**	24 (25.3%)	6 (21.4%)	18 (26.9%)	
** Normal**	24 (25.3%)	6 (21.4%)	18 (26.9%)	

Continuous variables are shown as median (1 quartile, 3 quartile). Categorical variables are shown as *n* (%). *P*-values were calculated with Fisher’s exact test for categorical variables and the Wilcoxon rank-sum test for continuous variables.

### Transcriptomic differences associated with sepsis mortality

**Figure 1:**
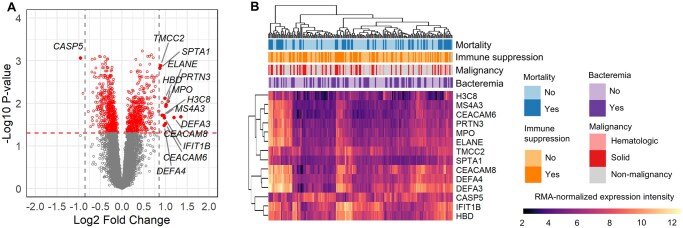
Differentially expressed genes related to sepsis mortality. (A) Volcano plot of differential expression results for sepsis nonsurvivors vs survivors in the MESSI cohort. Differentially expressed genes with BH-adjusted *P*-values <.05 are colored in red circles. A selected parsimonious gene set with 14 top-ranked protein-coding genes based on having BH-adjusted *P*-values <.05 and absolute log_2_FC ≥ 0.85 is annotated with their gene symbols (larger red dots). (B) Hierarchical clustering of the MESSI participants. Dendrograms correspond to hierarchical clustering based on Euclidean distance of RMA-normalized expression intensities of the 14 top-ranked genes (*y*-axis) across 161 individuals (*x*-axis). Color annotations representing each individual’s clinical status are shown as bars along the *x*-axis. RMA, robust multi-array average.

Comparison of gene expression levels between sepsis 30-day nonsurvivors vs survivors revealed 1106 DEGs in the MESSI cohort (Figure 1A and Table S2). The parsimonious gene set with 14 top-ranked differentially expressed protein-coding genes (ie, meeting absolute log_2_FC ≥ 0.85) is provided in Table 3. The defensin gene *DEFA3* had the greatest expression change, with a 2.6-fold increase in nonsurvivors. Seven genes (*CEACAM8*, *ELANE*, *PRTN3*, *MPO*, *CEACAM6*, *DEFA4*, *MS4A3*) were part of the *Reactome: ­neutrophil degranulation* pathway; by ImmuNexUT,^19^ each of these are exclusively expressed in neutrophils, except for *MPO*, which is also expressed in classical monocytes (Figure S2). The parsimonious DEG set also included *TMCC2* (encoding transmembrane and coiled-coil domain family 2) and *SPTA1* (encoding spectrin alpha, erythrocytic 1) with highly upregulated expression in nonsurvivors (BH-adjusted *P*-values = .001). The apoptosis-inducing gene *CASP5* was the only top-ranked protein-coding gene with decreased expression levels in nonsurvivors, with a 0.5-fold reduction (log_2_FC = −0.96). Unsupervised hierarchical clustering based on the expression levels of these 14 top-ranked genes yielded only partial differentiation between sepsis nonsurvivors and survivors (Figure 1B), and clustering all DEGs with a less stringent fold change requirement (absolute log_2_FC ≥ 0.5) looked similar (Figure S3A).

**Table 3:** Top-ranked differentially expressed genes in the comparison of 30-day sepsis nonsurvivors vs survivors in the MESSI cohort.

Gene symbol	Gene name	MESSI		MARS	
		Log_2_FC	BH-adjusted *P*-value	Log_2_FC	BH-adjusted *P*-value
***DEFA3***	Defensin alpha 3	1.35	.02	0.52	.03
***CEACAM8* ^a^**	CEA cell adhesion molecule 8	1.20	.02	0.79	.05
***ELANE* ^a^**	Elastase, neutrophil expressed	1.06	.009	0.80	.02
***PRTN3* ^a^**	Proteinase 3	1.02	.01	0.46	.02
***MPO* ^a^**	Myeloperoxidase	1.01	.01	0.70	.02
***CEACAM6* ^a^**	CEA cell adhesion molecule 6	1.00	.03	0.81	.03
***HBD***	Hemoglobin subunit delta	0.98	.008	0.58	.09
***IFIT1B***	Interferon induced protein with tetratricopeptide repeats 1B	0.97	.02	0.62	.12
***DEFA4* ^a^**	Defensin alpha 4	0.97	.03	0.98	.02
***MS4A3* ^a^**	Membrane spanning 4-domains A3	0.96	.02	0.92	.02
***H3C8***	H3 clustered histone 8	0.93	.02	0.09	.12
***TMCC2***	Transmembrane and coiled-coil domain family 2	0.88	.001	0.79	<.001
***SPTA1***	Spectrin alpha, erythrocytic 1	0.87	.001	0.46	<.001
***CASP5***	Caspase 5	−0.96	<.001	−0.48	.08

The parsimonious gene set with top-ranked differentially expressed genes (*N* = 14) was selected based on having BH-adjust *P*-values <.05 and absolute log_2_FC ≥ 0.85 (FC ≥1.80 or ≤0.56). Genes are sorted in descending order based on log_2_FC in the MESSI cohort.

a The neutrophil degranulation pathway genes.

### Replication and trans-cohort results

**Figure 2:**
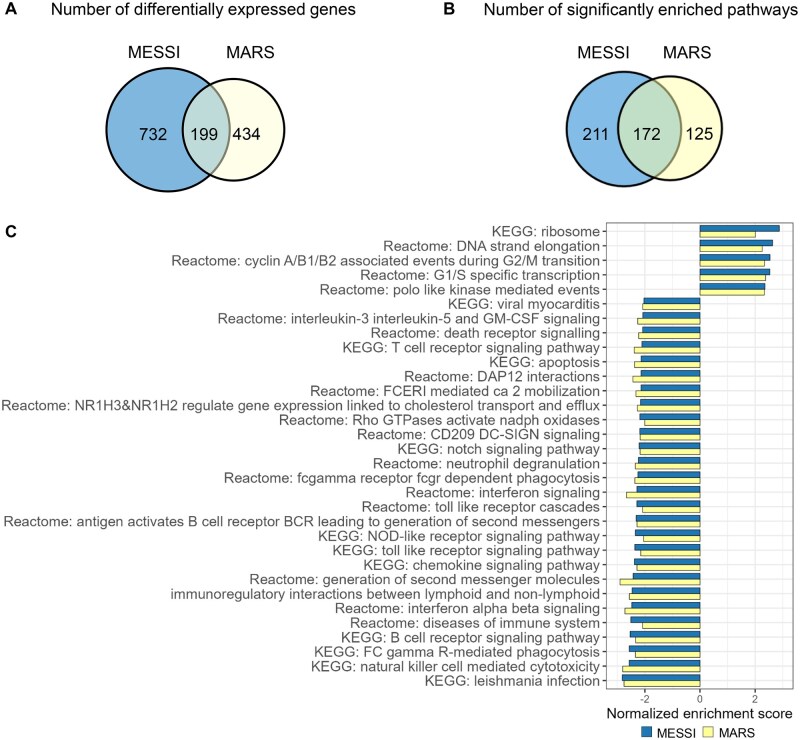
Transcriptomic differences associated with sepsis mortality in MESSI and MARS. Venn diagrams of (A) differentially expressed genes and (B) overrepresented pathways identified by GSEA based on the comparison of nonsurvivors vs survivors in MESSI (blue) and MARS (yellow). One gene with inconsistent log_2_ fold change signs and twelve pathways with inconsistent NES signs between the 2 cohorts, though significant, were not categorized as overlaps. (C) Barplots of shared top-ranked pathways corresponding to GSEA results of sepsis mortality. Thirty-two pathways were selected based on having BH-adjusted *P*-value <.05, absolute NES >2, and being designated as independent pathways using *fgsea* collapsedPathways function in both MESSI (blue) and MARS (yellow). Pathways are sorted by NES based on GSEA results from the MESSI dataset. NES, normalized enrichment score.

In the MARS cohort, there were 654 DEGs (BH-adjusted *P*-value <.05) when comparing 28-day nonsurvivors vs survivors. Comparing findings of 17 761 genes analyzed in both cohorts, 199 DEGs associated with sepsis mortality overlapped and showed consistent direction of expression changes, which we refer to as the trans-cohort gene set (Figure 2A and Table S3). Of the 14 top-ranked genes identified from MESSI (the parsimonious gene set), 9 were also identified as DEGs (BH-adjusted *P*-value <.05) in the MARS cohort: *DEFA3*, *TMCC2*, *SPTA1*, and 6 of the 7 neutrophil degranulation pathway genes (*ELANE*, *PRTN3*, *MPO*, *CEACAM6*, *DEFA4*, *MS4A3*) (Table 3). Hierarchical clustering using the trans-cohort gene set did not improve the separation of survivors and nonsurvivors compared with the parsimonious gene set from MESSI alone (Figure S3B), whereas the predictive model distinguishing survivors from nonsurvivors achieved a higher AUROC (0.71 for the trans-cohort gene set vs only 0.61 for the parsimonious gene set) (Figure S4).

Functional enrichment analysis was performed focusing on the trans-cohort gene set (199 genes). This analysis found that the *neutrophil degranulation* pathway genes were overrepresented in the trans-cohort gene set (16 of 199 genes, 8.0%) compared to their proportion in the background genes that were tested (439 of 17,761 genes, 2.5%) (BH-adjusted *P*-value = .03) (Table S4). Additionally, genes from 6 pathways related to Rho GTPase cycle regulation were overrepresented in the trans-cohort gene set (BH-adjusted *P*-value ≤.03). Among genes involved in these pathways, *ARHGEF12*, encoding a Rho guanine nucleotide exchange factor, exhibited the greatest expression change, with a 1.7-fold increase in MESSI nonsurvivors.

### GSEA-identified overrepresented pathways

Results of GSEA found 384 significantly overrepresented pathways associated with sepsis mortality in the MESSI dataset (BH-adjusted *P*-value <.05). Of these, 172 pathways were also significantly ­overrepresented in the MARS dataset, driven by genes with expression changes in the same direction as in the MESSI dataset, as indicated by concordance of normalized enrichment scores (NES) (Figure 2B and Table S5). Among them, 32 independent pathways were highlighted based on absolute NES >2 (Figure 2C), including pathways reflecting complex and myriad immune responses to pathogens (*Reactome: neutrophil degranulation*, *Reactome: interferon signaling*, *KEGG: chemokine signaling pathway*), as well as programmed cell death (*Reactome: death receptor signaling*, *KEGG: apoptosis*) and immune-mediated cytotoxicity (*KEGG: natural killer cell mediated cytotoxicity*). Interestingly, despite notable upregulation of neutrophil degranulation genes in nonsurvivors from the differential expression results, the overrepresentation of the neutrophil degranulation pathway was mainly driven by genes with *decreased* expression in nonsurvivors, including Fc gamma receptor genes (*FCGR2A*, *FCGR3B*) and chemokine receptor genes (*CXCR1*, *CXCR2*) that were significantly differentially expressed (BH-adjusted *P*-value <.05).

### Influence of infection and immune suppression on mortality-related expression differences

We found that the 14 top-ranked DEGs from MESSI (the parsimonious gene set) remained significant after adjusting for bacteremia or immunocompromised status in the 161 participants (Table S6). Notably, the highlighted pathways related to neutrophil degranulation, interferon signaling, chemokine signaling, death receptor signaling, and natural killer cell-mediated cytotoxicity all showed significant enrichment in GSEA results obtained from both pairwise comparison approaches, with consistent directionality (Table S7). Hierarchical clustering based on genes from either the parsimonious or trans-cohort gene set did not identify bacteremia or solid vs hematologic malignancy as being integral to cluster identity of nonsurvivors (Figure 1B and Figure S3).

### Gene co-expression associated with sepsis mortality and clinical phenotypes

**Figure 3:**
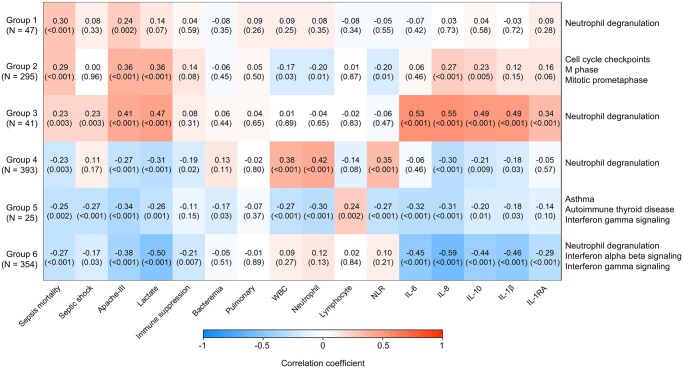
Heatmap of gene co-expression groups associated with sepsis mortality. Correlations were tested between *eigengenes* of identified gene co-expression groups and clinical variables in the MESSI dataset. Six groups are shown, selected because they exhibited (1) fewer than 500 genes and (2) at least a weak correlation with sepsis mortality (absolute Pearson correlation coefficient >0.20 and *P*-value <.05). Each box contains the correlation coefficient (top) and corresponding *P*-value (bottom) for a given group vs phenotype pair. Highlighted overrepresented pathways for each group are annotated on the right side of the heatmap. The color bar represents the scale of correlation coefficients. APACHE III, acute physiology and chronic health evaluation III score; IL, interleukin; IL1RA, interleukin-1 receptor antagonist; NLR, neutrophil-to-lymphocyte ratio; WBC, white blood cell.

WGCNA was performed on MESSI gene expression data for the shared 17,761 genes tested, where a parameter of soft-thresholding power (β) of 6 was chosen to generate an unsigned weighted co-expression network (Figure S5). Twenty-seven groups of co-expressed genes were identified. Among them, 6 groups were selected based on (1) having fewer than 500 genes and (2) demonstrating significant and at least weak correlation with sepsis mortality (Pearson correlation coefficient (*r*) >0.20 or <-0.20 and *P*-value <.05), shown in Figure 3, ordered by their correlation with sepsis mortality. Significant results from functional enrichment analysis, which assessed overrepresentation of *Reactome: neutrophil degranulation* (*P*-value <.05) and various other pathways (BH-adjusted *P*-value <.05) in each co-expression group, are listed in Table 4 and Table S8, respectively.

**Table 4:** Four gene co-expression groups with a significant overrepresentation of genes in the neutrophil degranulation pathway.

Co-expression group	Gene count	Percentage of gene count	*P*-value	BH-adjusted *P*-value	Genes
**Group 1 (*N* = 47)**	18	38.3	3.20 × 10^−16^	3.73 × 10^−13^	*ELANE* ^a^, *PRTN3* ^a^, *MPO* ^a^, *CTSG* ^a^, *MS4A3* ^a^, *CEACAM8* ^a^, *CEACAM6* ^a^, *DEFA4* ^a^, *AZU1* ^a^, *ABCA13*, *CAMP*, *OLR1*, *PRG3*, *PTX3*, *RNASE3*, *SERPINB10*, *SLC2A5*, *TARM1*
**Group 3 (*N* = 41)**	13	31.7	1.34 × 10^−10^	1.56 × 10^−7^	*CRISP3* ^a^, *CLEC5A*, *ENPP4*, *GPI*, *LCN2*, *LTF*, *MMP8*, *OLFM4*, *PLD1*, *PSMB7*, *RETN*, *STOM*, *TCN1*
**Group 4 (*N* = 393)**	23	5.9	3.22 × 10^−4^	.36	*FCGR2A* ^a^, *CXCR2* ^a^, *HSPA6* ^a^, *ACP3* ^a^, *MANBA* ^a^, *CXCR1* ^a^, *RNASET2* ^a^, *CPPED1* ^a^, *RAC1* ^a^, *FCGR3B* ^a^, *CD93*, *FRK*, *FTH1*, *HPSE*, *IRAG2*, *LAMP2*, *MAPK1*, *MGAM*, *MNDA*, *PAFAH1B2*, *PECAM1*, *QPCT*, *SLPI*
**Group 6 (*N* = 354)**	20	5.6	1.32 × 10^−3^	.12	*HLA-A* ^a^, *SLC15A4* ^a^, *CD300A* ^a^, *HVCN1* ^a^, *CRISPLD2* ^a^, *CTSZ* ^a^, *ADGRE3*, *ARHGAP45*, *CLEC4C*, *CRACR2A*, *GHDC*, *HLA-B*, *HLA-C*, *IGF2R*, *KPNB1*, *MME*, *PDXK*, *PLAUR*, *PSAP*, *RAB37*

Significance is based on a *P*-value <.05 from functional enrichment analysis. Gene count corresponds to the number of genes within the co-expression group that are part of *Reactome: neutrophil degranulation* pathway. The percentage of gene count corresponds to the percentage that the gene count represents of the total number of genes in the co-expression group. Genes are ordered by their adjusted *P*-values based on the differential expression results for sepsis mortality from the MESSI cohort.

a Differentially expressed genes with BH-adjusted *P*-value <.05 in the comparison of 30-day sepsis nonsurvivors vs survivors.

All 6 gene co-expression groups were associated with APACHE III scores, and 5 of the 6 groups were associated with lactate (*P*-value <.05), all with concordant directionality as their correlation with mortality. Four of the 6 groups (Group 1, 3, 4, 6) had genes overrepresented in the neutrophil degranulation pathway (*P*-value <.05). Group 1, which showed the strongest correlation with increased mortality risk, contained 9 differentially expressed neutrophil degranulation genes upregulated in nonsurvivors (*ELANE*, *PRTN3*, *MPO*, *CTSG*, *MS4A3*, *CEACAM8*, *CEACAM6*, *DEFA4*, *AZU1*), whereas Group 4, correlated with decreased mortality risk, contained 10 differentially expressed neutrophil degranulation genes downregulated in nonsurvivors (*FCGR2A*, *CXCR2*, *HSPA6*, *ACP3*, *MANBA*, *CXCR1*, *RNASET2*, *CPPED1*, *RAC1*, *FCGR3B*), which were among genes driving the overrepresentation of this pathway in GSEA results (Table S5). Interestingly, co-expression of genes in Group 4 was correlated with increased white blood cell counts, neutrophil counts, and neutrophil-lymphocyte ratio, whereas individual DEGs’ associations with mortality were independent of neutrophil counts. Groups 3 and 6 contained 1 upregulated (*CRISP3*) and 6 downregulated DEGs (*HLA-A*, *SLC15A4*, *CD300A*, *HVCN1*, *CRISPLD2*, *CTSZ*) from the neutrophil degranulation pathway, respectively. Group 6 also had genes overrepresented in pathways related to interferon signaling (the *Reactome: interferon alpha beta signaling*, *Reactome: interferon gamma signaling*), which includes the major histocompatibility complex class I gene *HLA-A* as a part of the pathway.

Co-expression Groups 2 and 5 did not have an overrepresentation of genes in neutrophil degranulation pathway but instead had an overrepresentation in other pathways, including those ­related to cell cycle (eg, *Reactome: cell cycle checkpoints*, *Reactome: M phase*, *Reactome: mitotic prometaphase*) in Group 2 and those related to diseases involving the immune system (eg, *KEGG: asthma*, *KEGG: autoimmune thyroid disease*) and interferon signaling (*Reactome: interferon gamma signaling*) in Group 5. Additionally, Groups 2 and 5 were correlated with decreased white blood cell counts, neutrophil counts, and neutrophil-lymphocyte ratio (*P*-value <.05).

For each of the 6 groups identified with MESSI data, we computed *eigengenes* using MARS gene expression data. The 3 groups that were positively correlated with mortality in MESSI (Groups 1-3) were significantly associated with 28-day sepsis mortality in MARS (*P*-value <.05) (Table S9).

### Plasma cytokines associate with mortality-associated gene co-expression groups

Cytokine measurements are shown in Table S10. Although none of the genes encoding the 5 measured cytokines were within the 6 gene co-expression groups, we observed that 5 of the 6 groups (Groups 2-6) were correlated with cytokine expression levels (Figure 3). Two groups showed weak-to-moderate association (Pearson *r* >0.20 or <−0.20): Group 3 was associated with higher cytokine expression levels and increased sepsis mortality risk, whereas Group 6 was associated with lower cytokine expression levels and decreased sepsis mortality risk. Associations with cytokines displayed concordant directionality as the co-expression groups’ correlation with mortality risk.

## Discussion

Our analysis of peripheral whole blood transcriptomes of 161 patients with sepsis at the time of ICU admission found many significant expression changes associated with 30-day sepsis mortality, including neutrophil degranulation pathway genes such as *CEACAM8*, *ELANE*, *PRTN3*, *MPO*, *CEACAM6*, *DEFA4*, and *MS4A3*, with increased expression in nonsurvivors and *FCGR2A*, *FCGR3B*, *CXCR2*, and *CXCR1* with decreased expression. In WGCNA co-expression analyses, these 2 groups of genes were members of co-expression groups associated with increased and decreased, respectively, risk of sepsis mortality. Comparison of MESSI findings to those of 28-day sepsis mortality in MARS suggests that the MESSI findings are generalizable: the top-ranked genes and pathways, such as those involved in neutrophil degranulation and interferon signaling, demonstrated consistent mortality associations. Furthermore, both the individual genes and pathways we identified replicate signals identified in other transcriptomic evaluations of sepsis outcomes. At the gene level, *PRTN3*, *MPO*, *CEACAM8*, *DEFA4*, *and SPTA1* from our parsimonious gene set, along with genes overrepresented in our mortality-associated co-expression groups (*LCN2*, *OLFM4*, *RETN*, *TCN1* in high mortality risk Group 3, and *FCGR2A*, *CXCR1*, *and CXCR2* in our low mortality risk Group 4), are consistent with prior reports.^24-27^ In addition, the correlations that we observed between co-expression Groups 3 and 6 with plasma cytokines in opposing directions are highly consonant with the gene expression findings reported in “reactive” and “uninflamed” ARDS subphenotypes.^25^

Dysregulated neutrophil activity has been widely reported in sepsis-related disease and death,^28-32^ a feature reproduced in whole blood transcriptomics of pediatric sepsis.^32^ Functional assays of neutrophils during sepsis have demonstrated a preponderance of immature neutrophils, which is consonant with activation of emergency granulopoiesis as a response to pathogens.^33^ The identification of the upregulation of azurophilic granule genes in nonsurvivors (*MPO*, *ELANE*, *CEACAM8/6*, *PRTN3*) suggests the presence of the immature neutrophil phenotype. This aligns with recent transcriptomic studies, both single-cell and bulk deconvolution, that describe expansion of an immature subpopulation with comparable gene expression profiles involving *CEACAM8*, *MPO*, and *IL1R2.*^34^^,^^35^ These reports support a shift toward emergency granulopoiesis and away from lymphopoiesis as contributing to immune dysregulation during sepsis.^34^ Sepsis neutrophils demonstrate impaired chemotaxis and oxidative bursts,^36^^,^^37^ which aligns with the decreased expression of chemokine receptor genes (*CXCR1*, *CXCR2*) observed in nonsurvivors. Moreover, we demonstrate overrepresentation of interferon signaling pathways in genes with decreased expression in sepsis nonsurvivors as well as in 2 co-expression groups correlated with decreased sepsis mortality. Consistent with this observation, single-cell sequencing of peripheral blood mononuclear cells revealed monocyte clusters exhibiting both suppression of genes responding to type-I interferon and upregulated gene targets associated with neutrophil degranulation that distinguished severe COVID-19 cases compared to non-severe cases.^38^ Taken together, these results suggest that the expansion of immature degranulating neutrophils in response to emergency granulopoiesis may serve as a cellular biomarker for high mortality risk in sepsis. Additionally, the identification of cell-specific expression quantitative trait loci (eQTL) in neutrophil-specific genes such as *PRTN3*, *DEFA4*, and *MS4A3*^19^ suggests they may contribute to individual variation in sepsis mortality risk via genetic variations that influence their expression in target cells.

We acknowledge that the mortality-associated expression signatures may be influenced by other clinical factors, such as specific pathogens and immunocompromising conditions. Although we observed overrepresentation of several pathways, including interferon signaling pathways, death receptor signaling, and natural killer cell-mediated cytotoxicity, which may suggest an anti-viral immune response, only 7% of our study population had viral sepsis. In addition, 7 of the 12 participants with a documented viral infection had bacterial co-infection, hampering our ability to compare bacterial- and viral-infected participants. We therefore focused on the influence of bacteremia as a proxy for pathogen burden, as we observed an excess of bacteremia among nonsurvivors. We also asked whether bacteremia or immune compromise was responsible for the individual genes or pathways associated with mortality. Our adjusted analyses yielded almost identical results at the individual gene and pathway level, with consistent enrichment of neutrophil degranulation and interferon signaling, suggesting neither bacteremia nor immune suppression explains our findings. To further subphenotype our immune suppression subgroup, we subdivided participants into those with non-cancer immunosuppression, solid malignancy, or hematologic malignancy, which we have demonstrated to modify certain molecular associations during sepsis.^16^^,^^23^ Hierarchical clustering did not reveal subgroups grouped according to bacteremia or specific immunocompromising condition, which we interpret as these ­factors being unlikely to explain our observed mortality-associated expression signatures.

Given the availability of numerous clinical characteristics in the MESSI cohort and the large number of DEGs identified, we applied WGCNA to identify smaller groups of gene co-expression groups associated with specific clinical characteristics that might provide context for our results. By focusing on genes with expression data available in both MESSI and MARS, we were able to assess the reproducibility of the identified co-expression groups in the MARS dataset. We found that 3 of the 6 groups correlated with increased risk of 30-day mortality in the MESSI cohort were also correlated with increased risk of 28-day sepsis mortality in the MARS cohort, suggesting that genes upregulated in nonsurvivors exhibit a similar pattern across both datasets. The co-expression groups correlated with sepsis mortality were also correlated with APACHE III scores and lactate, which are well-established indicators of severity of illness and mortality risk for critically ill patients.^39^^,^^40^ Neutrophil degranulation pathway genes were overrepresented in 4 of the 6 groups, suggesting that the effect of a group of genes from the pathway has a broader impact on sepsis mortality than individual genes. Of note, among 3 of the co-expression groups correlated with neutrophil counts, only 1 had genes overrepresented in the neutrophil degranulation pathway, highlighting that neutrophil degranulation signals are distinct from neutrophil abundance during sepsis.

We acknowledge that transcriptomic results from whole blood, particularly the neutrophil-related genes identified, do not always correspond directly to the actual proteins produced and released in blood. No white blood cell lysate was collected in this study, thus we focused on 5 plasma cytokines (IL-6, IL-8, IL-10, IL-1β, and IL-1RA), which are secreted from white blood cells in addition to other cell types and which we had measured in most of the gene expression subcohort. We examined their relationship with gene co-expression groups to bridge gene expression and protein expression while acknowledging that the correlation between mRNA and tissue protein abundance is highly variable.^41^ Two prominent co-expression groups (Groups 3 and 6), which showed moderate correlation with the expression levels of all the 5 cytokines, did not contain any of the parsimonious DEGs, whereas Group 1, which contained the largest number of the parsimonious gene set, was not associated with any of the cytokines. This suggests that the associations with cytokines are not directly linked to the mortality-associated genes with the largest expression changes. Although IL-8, as a pro-inflammatory cytokine, functions as a neutrophil chemotactic, whereas IL-10, as an anti-inflammatory cytokine, acts to suppress neutrophil activity, their correlation with gene co-expression groups was in the same direction, suggesting coordinated immune regulation in sepsis. For gene co-expression groups associated with cytokine expression, the direction of correlation was consistent with mortality risk. However, we acknowledge that plasma contains protein from different sources beyond white blood cells, which may affect circulating cytokine levels and complicate direct interpretation of their relationship with gene expression. Future work integrating cell-type-specific proteomic and transcriptomic data will be important to clarify the mechanistic roles of neutrophils in sepsis mortality.

We acknowledge several limitations to our study. First, because enrollment was required on ICU day 0 and we sought prospective consent to preserve mRNA compared to using residual day 0 plasma with delayed consent in the parent cohort, the gene expression subcohort was only a fraction of MESSI enrolled patients. Our cohort is uniquely enriched with patients suffering from septic shock and immune suppression, reflecting our hospital’s status as a quaternary center. Our broad eligibility criteria, which did not exclude participants with specific comorbidities, contributed to this enrichment. These factors may limit generalizability to a less severely ill sepsis population. Nevertheless, we showed that the gene expression subcohort was largely representative of the overall enrolled participants, and that mortality-associated gene expression changes persisted with adjustment for neutrophil count, bacteremia, and immune suppression. We further replicated these genes in an external cohort, highlighting the potential significance and reproducibility of our findings. Second, we assayed blood at the earliest available time point (ICU Day 0), but we acknowledge that sepsis is temporally dynamic and using a single time point in the continuum is insufficient. To fully understand and realize the diagnostic or prognostic possibilities of transcriptional data, a comprehensive time series to evaluate the changing transcriptome would be invaluable. Third, although we selected whole blood for its accessibility and practicality as a potential enrichment tool, the cellular origin of different transcriptional signals is not always certain and may limit translation to therapies. Future efforts could aim to disentangle these changes by performing single-cell analyses and explore whether the dysregulated pathways described here are modifiable or play a causal role in sepsis outcomes, and whether signatures derived from whole blood transcription can serve as treatment response biomarkers. Last, we acknowledge that gene expression microarrays have certain limitations compared to bulk RNA-sequencing, such as their inability to detect alterative splicing events and novel transcripts. However, microarrays remain a robust and cost-effective tool for transcript quantification in large population-based studies, and especially offer reliable quantification of known protein-coding transcripts, which aligns well with the scope of our study to identify genes associated with sepsis mortality. Future studies utilizing RNA-sequencing may capture a broader spectrum of transcriptomic variation, particularly in relation to novel or non-coding transcripts.

In summary, we performed whole blood transcriptomic profiling on 161 sepsis patients from the MESSI cohort to identify gene expression changes associated with sepsis mortality and severity. We compared our results to data from an independent cohort, with 479 sepsis patients with mortality data, to obtain findings that were reproducible across cohorts. Significant gene expression changes, particularly in neutrophil-specific genes, were associated with sepsis mortality. Our results highlight the involvement of neutrophil degranulation genes in sepsis mortality. These findings suggest an expansion of immature neutrophils in response to emergency granulopoiesis associated with sepsis mortality, though further investigation is needed to understand the underlying mechanisms driving this mortality.

## Supplementary Material

aanag021_Supplementary_Data
