# Supplementary material for: Whole blood transcriptomics reveals sepsis mortality-associated changes in neutrophil degranulation
Source: Am J Respir Cell Mol Biol. 2026 Feb 21;74(7):949–60. doi: 10.1093/ajrcmb/aanag021 (PMC13316924; doi:10.1093/ajrcmb/aanag021)
Supplement: aanag021_Supplementary_Data [file aanag021_supplementary_data.zip › Sepsis_AJRCMB_online_supplement_R1_101225.docx]

Whole blood transcriptomics reveals sepsis mortality-associated changes in neutrophil degranulation

# Online Supplement

**Table of Contents**

[Online Supplement 1](#_Toc211265704)

[Detailed Methods 3](#_Toc211265705)

[Table E1. Comparison of demographic and clinical characteristics between the overall MESSI cohort and the gene expression subcohort. 13](#_Toc211265706)

[Table E2. Differentially expressed genes identified in MESSI. 14](#_Toc211265707)

[Table E3. Trans-cohort differentially expressed genes. 14](#_Toc211265708)

[Table E4. Functional enrichment analysis of the 199 trans-cohort gene set associated with sepsis mortality. 14](#_Toc211265709)

[Table E5. GESA results corresponding to sepsis mortality. 15](#_Toc211265710)

[Table E6. Differential expression results accounting for the influence of clinical status. 16](#_Toc211265711)

[Table E7. GSEA results accounting for the influence of clinical status. 17](#_Toc211265712)

[Table E8. Functional enrichment analysis of gene co-expression groups. 18](#_Toc211265713)

[Table E9. Validation of associations between gene co-expression groups and sepsis mortality. 19](#_Toc211265714)

[Table E10. Cytokine levels in a subset of 151 participants. 20](#_Toc211265715)

[Figure E1. Study enrollment. 21](#_Toc211265716)

[Figure E2. Gene expression levels of top-ranked neutrophil degranulation pathway genes in neutrophils. 22](#_Toc211265717)

[Figure E3. Hierarchical clustering of the MESSI participants. 25](#_Toc211265718)

[Figure E4. ROC curves of predictive models distinguishing survivors and nonsurvivors. 26](#_Toc211265719)

[Figure E5. Selection of soft-thresholding power (β) for WGCNA. 27](#_Toc211265720)

# Detailed Methods

## Study Population and Clinical Phenotyping

This study was approved by the Institutional Review Board at the University of Pennsylvania (IRB #808542). Patients were enrolled in the Molecular Epidemiology of SepsiS in the ICU (MESSI) cohort at the Hospital of the University of Pennsylvania as previously described (1). Briefly, patients were eligible for enrollment if the primary cause for ICU admission was infection-related organ dysfunction consistent with Sepsis-3. Patient screening, recruitment, and enrollment occurred between November 2013 and April 2019, during which 7,554 ICU admissions were screened, and 1,773 patients were consented and enrolled (Figure E1). Exclusion criteria were applied to patients 1) if they were unable or declined to provide informed consent, lacked a reasonable proxy for consent, or with an exclusive focus on palliation, or 2) if they enrolled with only residual blood samples, if a fresh blood sample could not be collected within 24 hours or if they were leukopenic (white blood count <1000 cells/µL). Participants were enrolled with a waiver of timely informed consent allowing for early blood collection and informed consent was obtained from all patients or surrogates. After filtering,161 patients had whole blood RNA collected for the gene expression study.

Clinical data were collected on standardized report forms. Mortality was defined as death occurring within 30 days after ICU admission. Shock was defined as early (day 0-1) receipt of vasoactive medications. Immune suppression was defined as presence of metastatic malignancy, receipt of chemotherapy, immune modulators or high dose steroids, and/or presence of organ transplant. Demographic and clinical characteristics were compared between participants within and outside the MESSI gene expression subcohort, as well as between survivors and nonsurvivors, using Fisher’s exact test for categorical variables and Wilcoxon rank-sum test for continuous variables. P-values <0.05 were considered significant.

## Gene Expression Microarray and Data Analysis

Peripheral whole blood was collected in PAXgene™ RNA tubes (PreAnalytix, Hilden, Germany), and RNA was isolated via PAXgene Blood miRNA kit (Qiagen) per protocol. RNA quality was measured via Agilent Bioanalyzer 2100 (Agilent Technologies, Santa Clara, CA) and only samples with RNA Integrity Number >5 were included. A total of 161 participant samples (Figure E1) underwent genome-wide transcriptomic profiling in a single batch on Affymetrix Human Genome ST 2.1 arrays (Affymetrix, Sta. Clara, CA, USA). Raw intensity files in .CEL format were obtained and analyzed using the RAVED pipeline (<https://github.com/HimesGroup/raved>) (2). In brief, this pipeline included a quality control step based on the R package *arrayQualityMetrics* (3) and processing of raw intensities using the robust multi-array average (RMA) algorithm implemented in the R package *oligo* (4). Pairwise comparisons were made to assess expression differences in nonsurvivors versus survivors, with age, sex and neutrophil count included as covariates using the R package *limma* (v3.34.9) (5). The Benjamini-Hochberg (BH) approach was used to adjust for multiple comparisons and BH-adjusted p-values <0.05 were considered significant. Microarray probes were annotated to official gene symbols using the R array annotation package *hugene21sttranscriptcluster.db*. If multiple probes were annotated to the same gene symbol, the one with the smallest p-value and the largest absolute fold change was retained. The transcriptomic data and its analysis results are deposited in the Gene Expression Omnibus (GEO) under accession number GSE272769.

To highlight genes with the most notable expression changes, we applied a threshold based on BH-adjusted p-value <0.05 and absolute log_2_ fold change (FC) ≥0.85 (i.e., FC ≥1.80 or ≤0.55) and identified a parsimonious gene set with 14 protein-coding genes from the MESSI cohort, which we found to balance clarity and biological significance. This threshold allowed for a focused yet informative list of genes to present in the main text. All differentially expressed genes with BH-adjusted p-values <0.05, regardless of fold change, are provided in Table E2.

To account for potential influence of infection or immunosuppression on mortality-associated genes, we repeated the pairwise comparisons, additionally adjusting for bacteremia or immunocompromised status.

## Replication Cohort

To test whether our results generalized to an independent cohort, we obtained and analyzed transcriptomic data from the Molecular Diagnosis and Risk Stratification of Sepsis (MARS) project, available in GEO under accession GSE65682. MARS is a prospective observational cohort of adult patients with sepsis admitted to the ICU in two academic hospitals in the Netherlands between January 2011 and July 2013 (6). Patient samples were collected within the first 24 hours of admission to ICU and survival was monitored for 28 days. Gene expression microarray data in .CEL format and the GEOMatrix file were downloaded from GEO. Phenotype information was extracted from the GSEMatrix file. Gene expression data of 479 subjects with sepsis mortality information available were analyzed using the RAVED pipeline (2) as described for the MESSI dataset. Differential expression analysis compared expression changes in sepsis nonsurvivors versus survivors at 28 days, with age and sex included as covariates. Microarray probes were annotated to gene symbols using the R array annotation package *hgu219.db*. A trans-cohort gene set of 199 overlapping differentially expressed genes (BH-adjusted p-value <0.05) and exhibiting consistent direction of expression changes from MESSI and MARS was identified.

## Retrieval of Cell-Specific Gene Expression Levels

Gene expression levels in neutrophils of select genes were retrieved from the Immune Cell Gene Expression Atlas from the University of Tokyo (ImmuNexUT) (<https://www.immunexut.org/>) (7).

## Hierarchical Clustering

Hierarchical clustering was performed using Ward’s method with Euclidean distance (8) to identify and cluster similarities across top-ranked differentially expressed genes and study participants according to sepsis mortality. Dendrograms were created based on the Euclidean distance between RMA-normalized gene expression intensity of three selected gene set: 1) the parsimonious gene set with 14 top-ranked genes from MESSI (BH-adjusted p-values <0.05 and absolute log_2_ FC ≥0.85), 2) all differentially expressed genes from MESSI with a less stringent fold change requirement (BH-adjusted p-values <0.05 and absolute log_2_ FC ≥0.5) and 3) the trans-cohort gene set with 199 overlapping differentially expressed genes from MESSI and MARS. The resulting clusters were visualized using R *pheatmap* package. Mortality status, immunocompromised status as well as malignancy types, and bacteremia status were included as grouping variables for color annotation.

## Predictive Model

Predictive models for sepsis mortality were developed from the two gene sets described above using LASSO logistic regression implemented in the R package *glmnet*. We fit the models to the RMA-normalized expression data of these genes from MESSI with 5-fold cross validation. The regularization parameter λ was selected from the sequence of λ values that yielded the smallest cross-validated mean squared error. Nineteen of the 199 genes (*RAP1GAP*, *CLEC4A*, *UBAC2*, *ITGAX*, *CANT1*, *PRTN3*, *ELANE*, *ARRDC2*, *HSPA13*, *TIMP3*, *PARVG*, *TNFSF10*, *EREG*, *AREG*, *TGFBI*, *FYB1*, *CDYL*, *ICA1*, *MAP7D2*) from the trans-cohort gene set and six of the 14 genes (*ELANE*, *PRTN3*, *H3C8*, *TMCC2*, *SPTA1*, *CASP5*) from the parsimonious gene set with non-zero coefficients were used in the predictive models. The performance of the predictive models was tested on the independent data, RMA-normalized expression data from MARS, and was evaluated using the Area Under the Receiver Operating Characteristic curve (AUROC) implemented in the R package pROC.

## Pathway Overrepresentation Analysis

Pathway overrepresentation analysis focused on KEGG and Reactome pathway annotation gene sets downloaded from MsigDB collections (v.7.4) (<http://software.broadinstitute.org/gsea/msigdb/collections.jsp>), including pathways with gene number >15 and <500 for analysis. Two approaches, functional enrichment analysis and gene-set enrichment analysis (GSEA), were used.

*Functional enrichment analysis.* For the 199 trans-cohort differentially expressed genes and the select WGCNA co-expression groups, functional enrichment analysis assessed whether genes from a specific pathway were overrepresented in *a gene set* *of interest* compared to their occurrence in the background genome, using a modified Fisher’s exact test, also known as the “EASE score” in the DAVID NIH resource (<https://david.ncifcrf.gov/helps/functional_annotation.html>) (9, 10). As *background* genes, we selected the 17,761 human genes tested in differential expression analysis whose gene symbols were available in both MESSI and MARS datasets. A 2×2 contingency table was constructed to compare the numbers of genes in a pathway that were also in a given gene set minus one (cell 1), not in in a given co-expression group (cell 2) versus the number of genes in the human genome that were in the pathway (cell 3) and not in the pathway (cell 4). A one-tailed Fisher’s exact test was then used to test whether genes in a given gene set were more likely to be enriched in the pathway compared to genes randomly selected from the human genome. The Benjamini-Hochberg approach was used to correct for multiple comparisons made (i.e., pathways considered) and adjusted p-values <0.05 were considered significant.

*GSEA*. GSEA identified pathways overrepresented in a ranked gene list, i.e., *all* the genes previously tested ordered by their differential expression results. GSEA was performed with the fast gene set enrichment analysis algorithm implemented in the *fgsea* R package (v.1.4.1) (11). Genes were sorted according to decreasing t-statistics from differential expression results obtained using the *limma* R package (12). If multiple probe IDs were annotated to the same gene symbol, the one with the largest absolute t-statistic was retained. Gene-level score for a gene in rank k was calculated by adding a positive or negative value, based on the presence or absence of the gene in the gene set, to the score of the gene in rank k-1, and positive values were weighted by gene k’s t-statistic. The enrichment score for a particular gene set was designated as the largest absolute value from all gene scores. P-values were assessed based on the distribution of enrichment scores following 10,000 permutations, and subsequently, adjusted p-values were obtained using the Benjamini-Hochberg approach. The leading-edge subsets, defined as genes in the ranked list that appear before and at the position of a positive gene-set enrichment score, or that appear at and after the position of a negative gene set enrichment score, corresponded to genes that drove the overrepresentation. Independent pathways were retained by collapsing dependent pathways using the *collapsedPathways* function in *fgsea*.

## Weighted Gene Co-expression Network Analysis

Weighted gene co-expression network analysis (WGCNA) was performed using the WGCNA R package (v.1.72) (13) to identify groups of genes with similar expression patterns and assess their correlation with various clinical phenotypes. Genes with expression data available in both the MESSI and MARS cohorts were included (N = 17,761). The expression matrix of selected genes was obtained based on robust multi-array average (RMA)-adjusted expression values.

The connection strength between genes was estimated using an unsigned weighted network *adjacency* (α), i.e., by rising Pearson correlation coefficients (s_ij_) to a power β: $\alpha_{ij}={|S_{ij}|}^{\beta}(\beta\geq1)$ for gene i and j, where β was selected using the scale-free topology criterion (14). The unsigned correlation network was constructed based on the assumption that negatively correlated genes have the same connection strength as the positively correlated ones. Network connectivity of the gene i (k_i_) was computed as the sum of the *adjacency* matrix with all the other genes (u) in the network: $k_{i}= \sum_{u\neq i} \alpha_{iu}$. The *topological overlap matrix* (TOM) between genes i and j was computed as: $TOM= \frac{\sum_{u\neq i,j} {\alpha_{iu}\alpha}_{uj}{+\alpha}_{ij}}{min(k_{i},k_{j})+1-\alpha_{ij}}$. Gene co-expression groups were defined as genes with high topologic overlap in expression and were determined as follows:

1) *Primary gene group determination*. Selected genes were clustered by hierarchical clustering of TOM-based dissimilarity matrix (i.e., 1-TOM matrix). The dynamic tree cut method was applied to cut the branches (i.e., genes) of the hierarchical tree, and genes within the same branch were combined into a group.

2) *Merging of highly similar groups*. Principal components of each gene co-expression group were computed using the corresponding *adjacency* matrix. The principal components were defined as *eigengenes* for each group and further used to determine group similarity. Groups were clustered by hierarchical clustering of 1-correlation matrix of *eigengenes*. Branches (i.e. gene co-expression groups) that had a height <0.25, corresponding to a correlation coefficient ≥0.75 were merged as final determined groups.

Correlation between the final groups and phenotypic variables was computed as Pearson correlation coefficients between *eigengenes* for each group and each phenotypic variable. The significance of correlation was computed using one-tailed student’s t-tests and p-values <0.05 were considered significant.

## Cytokine Measurement and Analysis

Plasma from 151 participants, a subset of the MESSI gene expression subcohort was collected into citrated vacutainers on the day of ICU admission (± 12 hours) (ICU day 0). Blood was centrifuged within 30 minutes of blood draw and kept at 4°C for 12–48 hours and then frozen at –80°C until analysis. Electrochemiluminescence (MesoScaleDiscovery, Meso Scale Diagnostics, Rockville, MD) was used to measure five plasma cytokines (interleukin (IL)-6, IL-8, IL-10, IL-1β, and IL-1 receptor antagonist (IL-1RA)) in multiplex as described (15). Natural logarithm transformation was performed on original cytokine concentrations plus 0.01 for normality. The log-transformed values were used to test for correlation with gene co-expression groups identified by WGCNA.

# **Table E1. Comparison of demographic and clinical characteristics between the overall MESSI cohort and the gene expression subcohort.**

| **Characteristics** | **Overall**  **(N = 1773)** | **Gene Expression subset (n=161)** | **No Gene Expression (n=1612)** | **P-value** |
| --- | --- | --- | --- | --- |
| Age (years) | 62 (52, 70) | 63 (53, 72) | 62 (52, 70) | 0.21 |
| Male | 1029 (58%) | 74 (46%) | 942 (58%) | 0.28 |
| Race / Ethnicity | | | | |
| White | 1133 (64%) | 99 (61%) | 1034 (64%) | 0.26 |
| Black | 518 (29%) | 54 (34%) | 464 (29%) |  |
| Asian | 69 (4%) | 6 (4%) | 63 (4%) |  |
| Other or More than one | 53 (3%) | 2 (1%) | 51 (3%) |  |
| Hispanic | 46 (3%) | 7 (4%) | 39 (2%) | 0.15 |
| APACHE III score | 86 (66, 112) | 84 (66, 114) | 86 (66, 112) | 0.74 |
| *Medical Comorbidities* | | | | |
| Immune Suppression | 982 (55%) | 74 (46%) | 908 (56%) | 0.011 |
| Diabetes Mellitus | 525 (30%) | 57 (36%) | 468 (29%) | 0.08 |
| Chronic Dialysis | 110 (6%) | 12 (7%) | 98 (6%) | 0.49 |
| *Sepsis Features* | | | | |
| Septic Shock (vasopressors) | 839 (47%) | 123 (76%) | 731 (45%) | <0.001 |
| Culture positive* | 1173 (66%) | 115 (71%) | 1058 (66%) | 0.12 |
| Primary source |  |  |  |  |
| Pulmonary | 871 (49%) | 60 (37%) | 810 (50%) | 0.01 |
| Gastrointestinal | 307 (17%) | 34 (21%) | 270 (17%) |  |
| Genitourinary | 181 (10%) | 21 (13%) | 159 (10%) |  |
| Bloodstream | 215 (12%) | 19 (12%) | 196 (12%) |  |
| Unclear | 109 (6%) | 14 (9%) | 101 (6%) |  |
| 30-day non-survival | 748 (42%) | 60 (37%) | 688 (43%) | 0.24 |
| Continuous variables are shown as median (1 quartile, 3 quartile). Categorical variables are shown as n (%). P-values were calculated with the Fisher’s exact test for categorical variables and Wilcoxon rank-sum test for continuous variables.  *Culture positive includes any microbiologically confirmed infection between day -7 to day +7 centered around ICU admission. All culture and molecular tests during this window were reviewed. | | | | |

# **Table E2. Differentially expressed genes identified in MESSI.**

Comparison of sepsis 30-day nonsurvivors versus survivors in MESSI revealed 1,106 differentially expressed genes (BH-adjusted p-value <0.05). Genes are sorted first in ascending order of BH-adjusted p-values and for ties, in descending order of absolute log_2_FC. The table is provided in a separate .xlsx file: TableE2_MESSI_DE_Genes.xlsx.

# **Table E3. Trans-cohort differentially expressed genes.**

The trans-cohort gene set contains 199 differentially expressed genes (BH-adjusted p-value <0.05) identified in both the MESSI and MARS cohorts. Genes are sorted in descending order based on log_2_FC in the MESSI cohort. The table is provided in a separate .xlsx file: TableE3_Transcohort_DE_Genes.xlsx.

# Table E4. Functional enrichment analysis of the 199 trans-cohort gene set associated with sepsis mortality.

The significance of pathways was assessed using functional enrichment analysis. Pathways with BH-adjusted p-values <0.05 are shown, along with the number of genes within the 199 trans-cohort gene set that were part of the pathway (Gene count), the names of these genes (Genes), the percentage that the gene count represents of the total number of genes in the 199 trans-cohort gene set (Percentage of gene count), and the corresponding adjusted p-value. The table is provided in a separate .xlsx file: TableE4_Functional_Enrichment_Analysis_Transcohort_Gene_Set.xlsx.

# Table E5. GESA results corresponding to sepsis mortality.

A total of 172 significantly overrepresented pathways (BH-adjusted p-value <0.05) identified in both the MESSI and MARS cohorts are shown, based on the ranking of differentially expressed genes when comparing sepsis nonsurvivors versus survivors. The genes in the leading edge are ordered by descending t-statistics based on the differential expression results from the MESSI cohort. NES: normalized enrichment score. The table is provided in a separate .xlsx file: TableE5_GSEA_Sepsis_Mortality.xlsx.

# **Table E6.** Differential expression results accounting for the influence of clinical status**.**

Differential expression results of 14 top-ranked genes from pairwise comparisons between nonsurvivors and survivors in 161 MESSI participants using three models: 1) with adjustment for age, sex, and neutrophil counts, and with adjustment for 2) bacteremia status and 3) immunocompromised status, respectively, along with age, sex, and neutrophil counts. Genes are sorted in descending order based on log_2_FC from the first model, matching the order presented in Table 3.

| Gene symbol | Without bacteremia/  immunocompromised status adjustment | | Bacteremia status adjustment | | Immunocompromised status adjustment | |
| --- | --- | --- | --- | --- | --- | --- |
|  | Log_2_FC | BH-adjusted p-value | Log_2_FC | BH-adjusted p-value | Log_2_FC | BH-adjusted p-value |
| *DEFA3* | 1.35 | y0.021 | 1.37 | 0.031 | 1.34 | 0.044 |
| *CEACAM8* | 1.20 | 0.022 | 1.21 | 0.031 | 1.21 | 0.040 |
| *ELANE* | 1.06 | 0.009 | 1.07 | 0.016 | 1.04 | 0.022 |
| *PRTN3* | 1.02 | 0.011 | 1.01 | 0.021 | 0.99 | 0.028 |
| *MPO* | 1.01 | 0.012 | 0.98 | 0.025 | 0.99 | 0.028 |
| *CEACAM6* | 1.00 | 0.030 | 1.03 | 0.036 | 1.01 | 0.053 |
| *HBD* | 0.98 | 0.008 | 0.94 | 0.020 | 0.92 | 0.026 |
| *IFIT1B* | 0.97 | 0.022 | 0.93 | 0.047 | 0.92 | 0.059 |
| *DEFA4* | 0.97 | 0.033 | 0.99 | 0.043 | 0.98 | 0.057 |
| *MS4A3* | 0.96 | 0.019 | 0.98 | 0.027 | 0.95 | 0.042 |
| *H3C8* | 0.93 | 0.019 | 0.86 | 0.046 | 0.89 | 0.047 |
| *TMCC2* | 0.88 | 0.001 | 0.85 | 0.005 | 0.85 | 0.006 |
| *SPTA1* | 0.87 | 0.002 | 0.85 | 0.005 | 0.79 | 0.011 |
| *CASP5* | -0.96 | 0.001 | -0.91 | 0.005 | -0.89 | 0.005 |

# **Table E7.** GSEA results accounting for the influence of clinical status**.**

GSEA results of eight selected pathways based on pairwise comparison results between nonsurvivors and survivors in 161 MESSI participants using three models: 1) with adjustment for age, sex, and neutrophil counts, and with adjustment for 2) bacteremia status and 3) immunocompromised status, respectively, along with age, sex, and neutrophil counts.

| Pathway | Without bacteremia/  immunocompromised status adjustment | | Bacteremia status adjustment | | Immunocompromised status adjustment | |
| --- | --- | --- | --- | --- | --- | --- |
|  | NES | BH-adjusted p-value | NES | BH-adjusted p-value | NES | BH-adjusted p-value |
| REACTOME_NEUTROPHIL_DEGRANULATION | -2.24 | 0.001 | -2.12 | 0.002 | -2.24 | 0.002 |
| REACTOME_INTERFERON_SIGNALING | -2.29 | 0.001 | -2.44 | 0.002 | -2.06 | 0.002 |
| REACTOME_INTERFERON_ALPHA_BETA_SIGNALING | -2.48 | 0.001 | -2.58 | 0.002 | -2.30 | 0.002 |
| REACTOME_INTERFERON_GAMMA_SIGNALING | -2.64 | 0.001 | -2.74 | 0.002 | -2.41 | 0.002 |
| KEGG_CHEMOKINE_SIGNALING_PATHWAY | -2.38 | 0.001 | -2.29 | 0.002 | -2.24 | 0.002 |
| REACTOME_DEATH_RECEPTOR_SIGNALLING | -2.08 | 0.001 | -2.05 | 0.002 | -2.06 | 0.002 |
| KEGG_APOPTOSIS | -2.14 | 0.001 | -2.00 | 0.002 | -2.16 | 0.002 |
| KEGG_NATURAL_KILLER_CELL_MEDIATED_CYTOTOXICITY | -2.57 | 0.001 | -2.44 | 0.002 | -2.54 | 0.002 |

# Table E8. Functional enrichment analysis of gene co-expression groups.

The significance of pathways was assessed using functional enrichment analysis. Significant overrepresented pathways (BH-adjusted p-value <0.05) other than *Reactome: neutrophil degranulation* in each of the six gene co-expression groups associated with sepsis mortality, along with the number of genes within the co-expression group that were part of the pathway (Gene count), the names of these genes (Genes), the percentage that the gene count represents of the total number of genes in the co-expression group (Percentage of gene count), and the corresponding p-value and adjusted p-value. Genes are ordered by their adjusted p-values based on the differential expression results for sepsis mortality from the MESSI cohort. Among the six co-expression groups, Groups 3 and 5 that lacked other overrepresented pathways (BH-adjusted p-value <0.05) are not shown in the Table. *Differentially expressed genes with BH-adjusted p-value <0.05 in the comparison of 30-day sepsis nonsurvivors versus survivors. The table is provided in a separate .xlsx file: TableE8_Fuctional_Enrichment_Analysis_Gene_Coexpression_Groups.xlsx.

# Table E9. Validation of associations between gene co-expression groups and sepsis mortality.

*Eigengenes* were computed for six gene co-expression groups associated with 30-day sepsis mortality identified from the MESSI cohort, based on their expression values in the MARS cohort. Pearson correlation coefficients were computed between *eigengenes* and 28-day sepsis mortality in the MARS cohort. The significance of correlations was assessed using one-tailed student’s t-tests. P-values <0.05 were considered significant.

| **Co-expression group** | **Pearson correlation coefficient** | **P-value** |
| --- | --- | --- |
| Group 1 | 0.13 | 0.004 |
| Group 2 | 0.14 | 0.002 |
| Group 3 | 0.12 | 0.009 |
| Group 4 | 0.14 | 0.002 |
| Group 5 | 0.12 | 0.008 |
| Group 6 | 0.14 | 0.003 |

# **Table E10. Cytokine levels in a subset of 151 participants.**

Original cytokine measurements are reported as median (1 quartile, 3 quartile).

| **Cytokine** | **Value(pg/mL)** |
| --- | --- |
| IL-6 | 176 (50, 1730) |
| IL-8 | 67 (22, 232) |
| IL-10 | 8 (2, 49) |
| IL-1β | 1.36 (0.39, 3.64) |
| IL-1RA | 2597 (588, 9493) |

# Figure E1. Study enrollment.

Patients were enrolled in MESSI cohort as described in methods and included in the nested MESSI gene expression cohort. Exclusion criteria were applied to patients if they were leukopenia, lack of fresh blood sample or inability to obtain timely sample within 24 hours of admission to ICU.


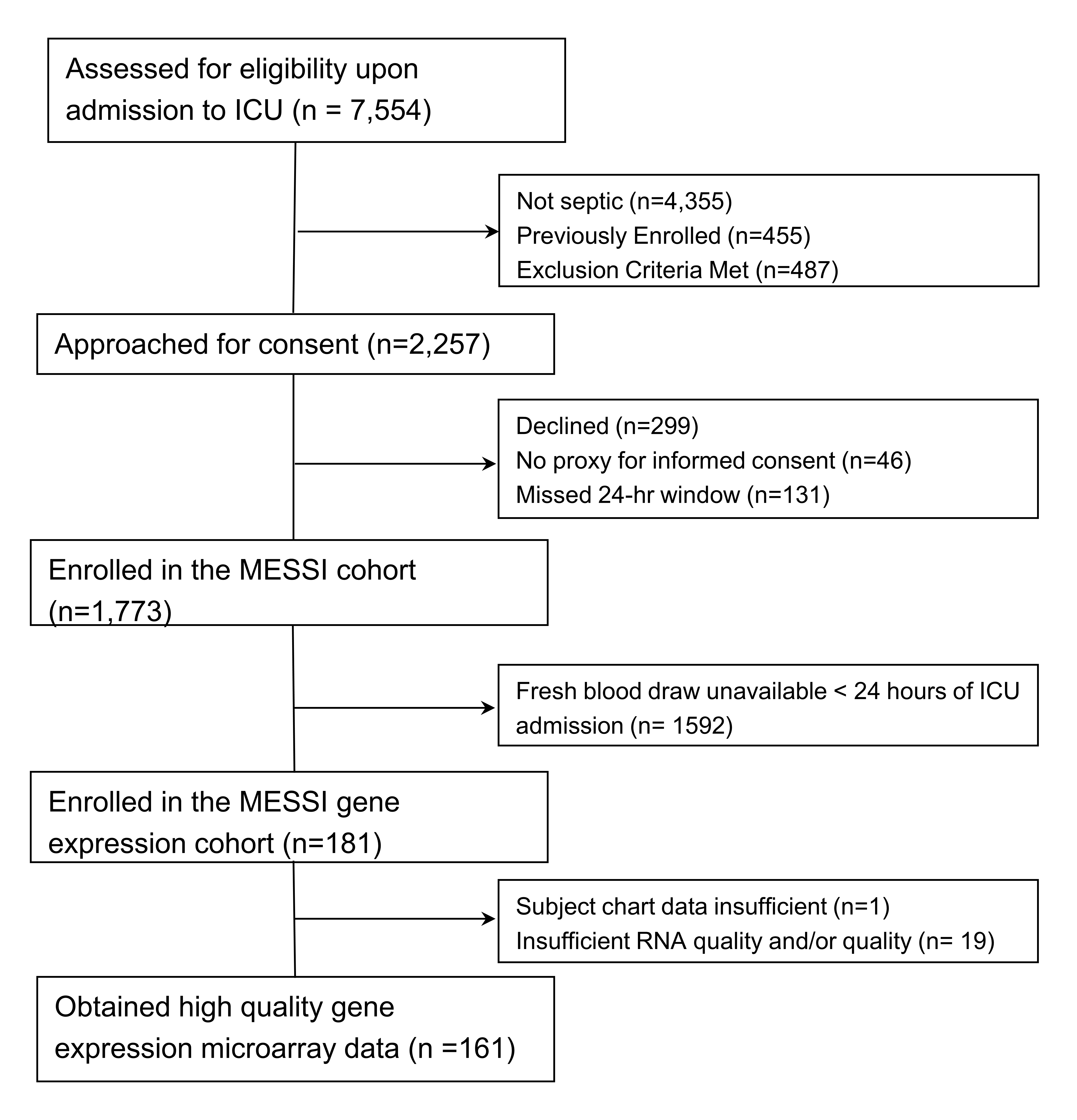


# Figure E2. Gene expression levels of top-ranked neutrophil degranulation pathway genes in neutrophils.

Boxplots of gene expression levels were retrieved from the Immune Cell Gene Expression Atlas from the University of Tokyo (ImmuNexUT) (<https://www.immunexut.org/>) (7). Naïve CD4: naïve CD4 T cells; Mem CD4: memory CD4 T cells; Th1: T helper 1 cells; Th2: T helper 2 cells; Th17: T helper 17 cells; Tfh: T follicular helper cells; Fr. I nTreg: fraction I naïve regulatory T cells; Fr. II eTreg: Fraction II effector regulatory T cells; Fr. III T: fraction III non-regulatory T cells; Naïve CD8: Naïve CD8 T cells; CM CD8: central Memory CD8 T cells; EM CD8: Effector Memory CD8 T cells; TEMRA CD8: CD8+ T effector memory CD45RA+ cells; NK: natural killer cells; Naïve B: Naïve B cells; USM B: unswitched memory B cells; SM B: switched memory B cells; DN B: double negative B cells; CL Mono: classical monocytes; CD16p Mono: CD16 positive monocytes; Int Mono: intermediate monocytes; NC Mono: non-classical monocytes; mDC: myeloid dendritic cells; pDC: plasmacytoid dendritic cells; Neu: neutrophils; LDG: low-density granulocyte; TPM: transcripts per million.


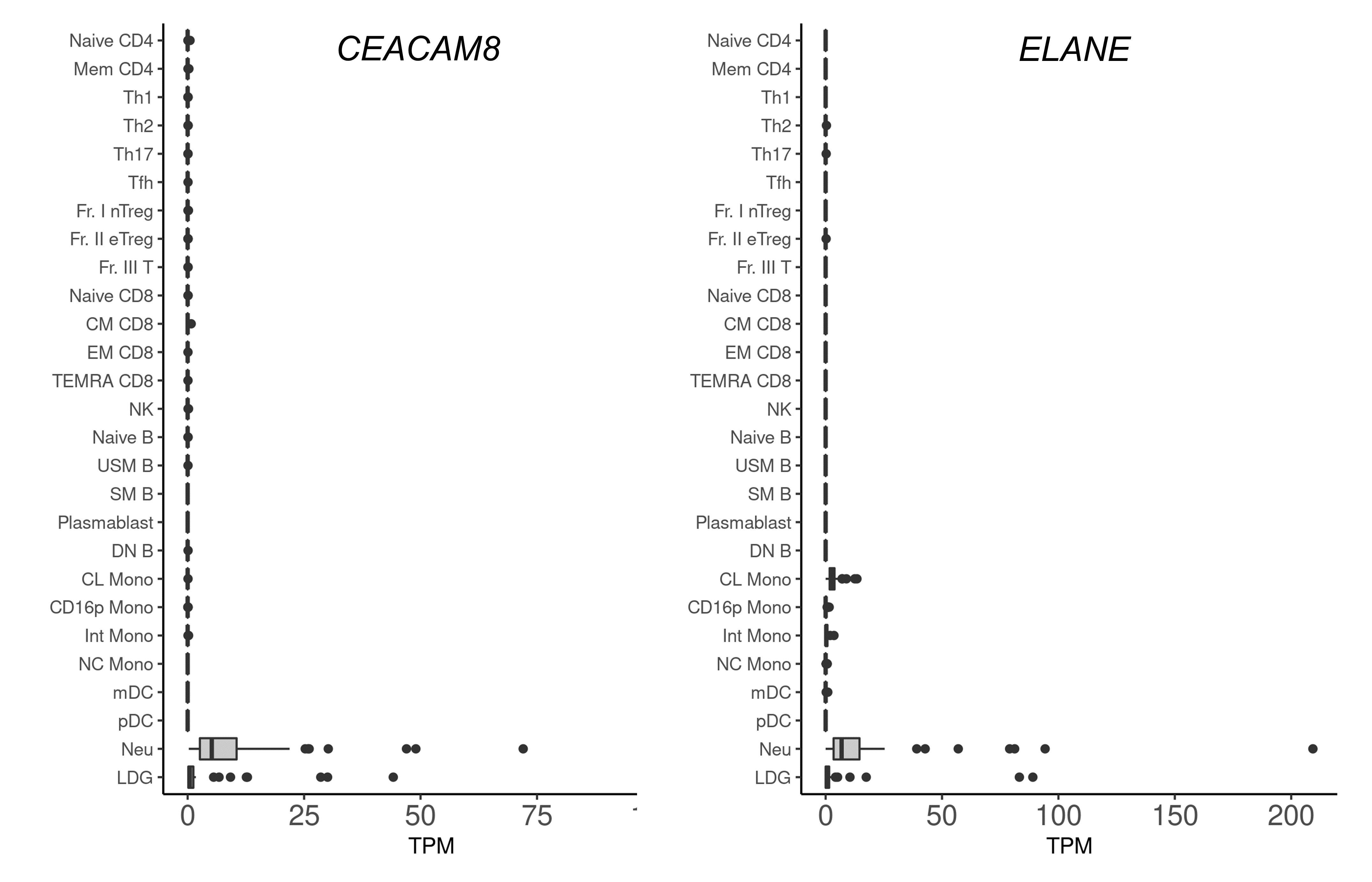


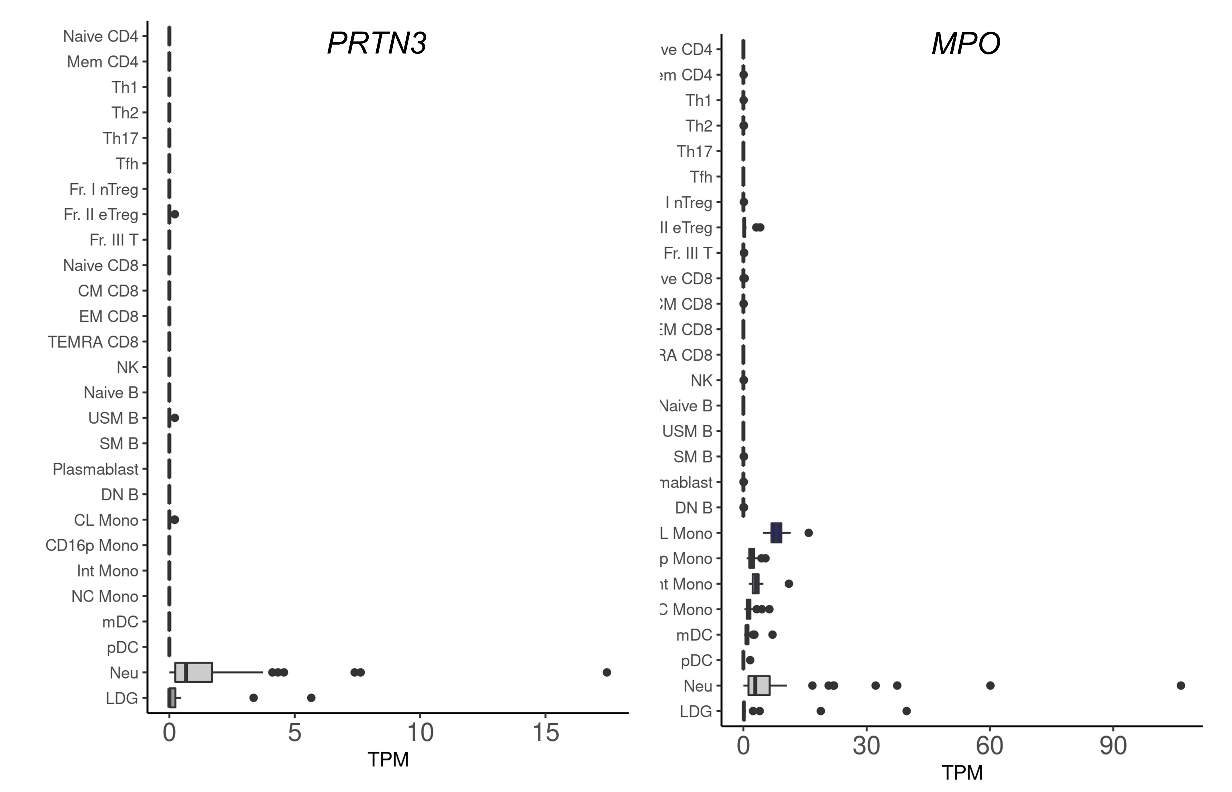


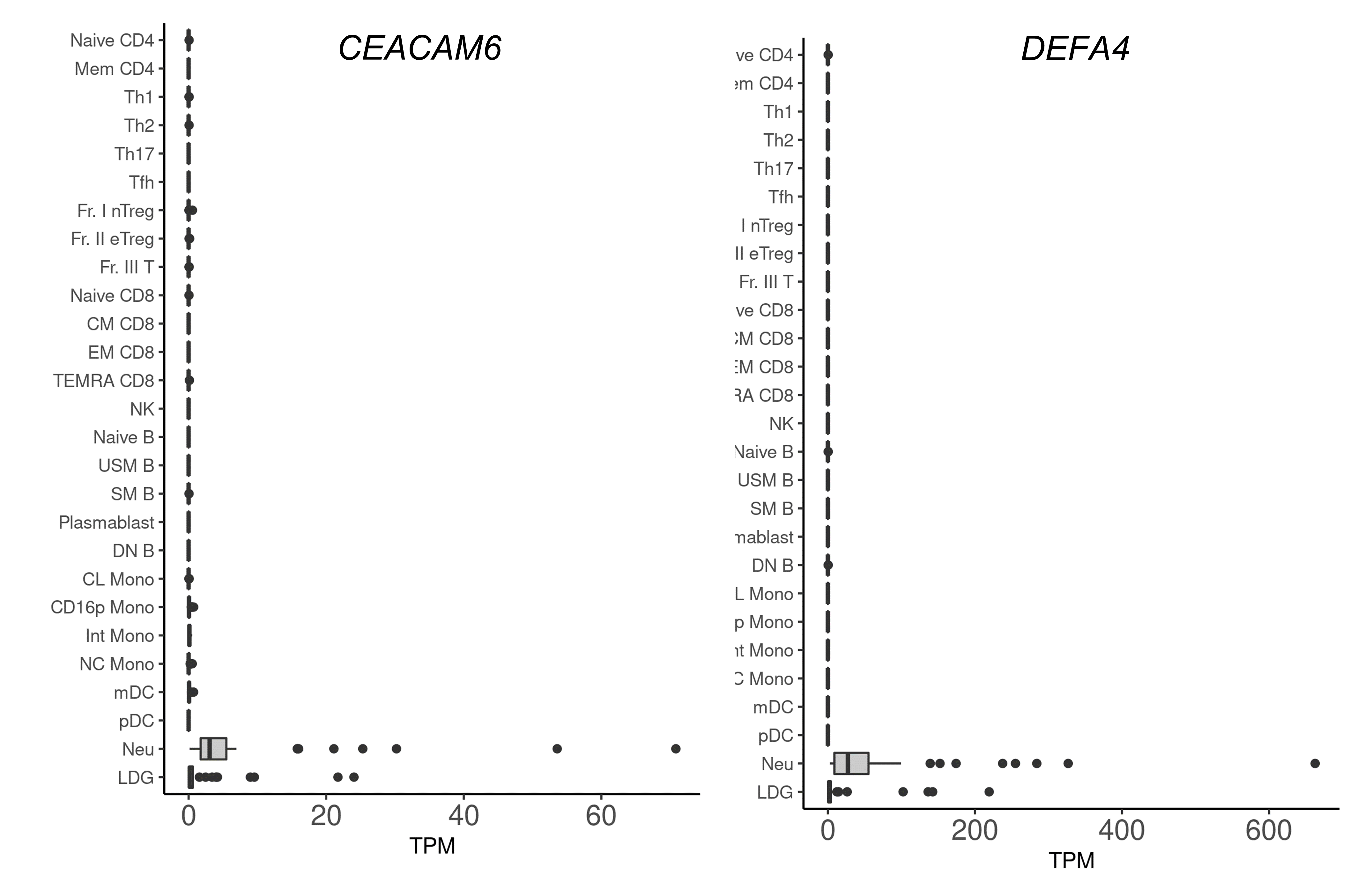


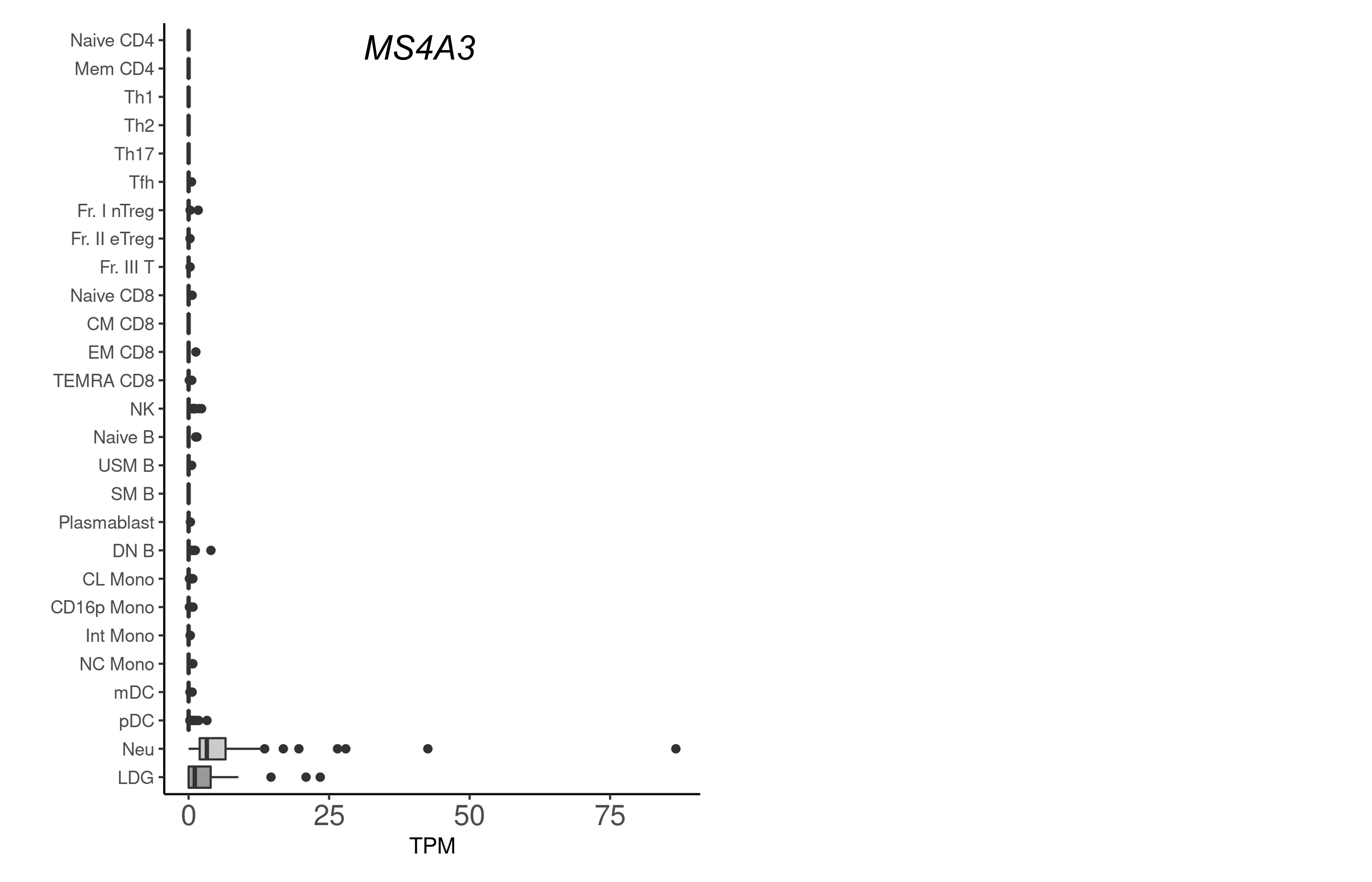


# Figure E3. Hierarchical clustering of the MESSI participants.

Hierarchical clustering was performed based on A) a gene set of 136 differentially expressed genes from MESSI (BH-adjusted p-value <0.05 and absolute log_2_FC ≥0.5) and B) a trans-cohort gene set with 199 differentially expressed genes (BH-adjusted p-value <0.05) from MESSI and MARS. Dendrograms correspond to hierarchical clustering based on Euclidean distance of RMA-normalized expression intensities of the selected differentially expressed genes (y-axis) across 161 MESSI participants (x-axis). Color annotations representing each individual’s clinical status are shown as bars along x-axis. RMA: robust multi-array average.


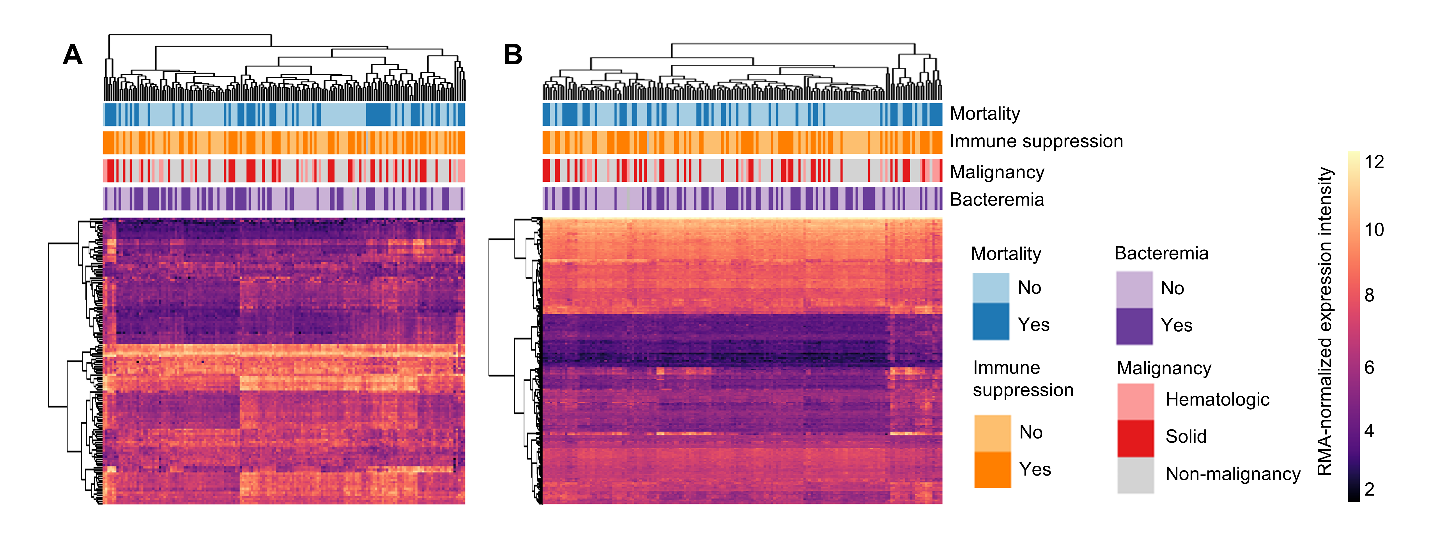


# Figure E4. ROC curves of predictive models distinguishing survivors and nonsurvivors.

Predictive models were created using A) the trans-cohort gene set with 199 overlapping differentially expressed genes from MESSI and MARS and B) the parsimonious gene set with 14 top-ranked genes from MESSI. AUROC: Area Under the Receiver Operating Characteristic curve.


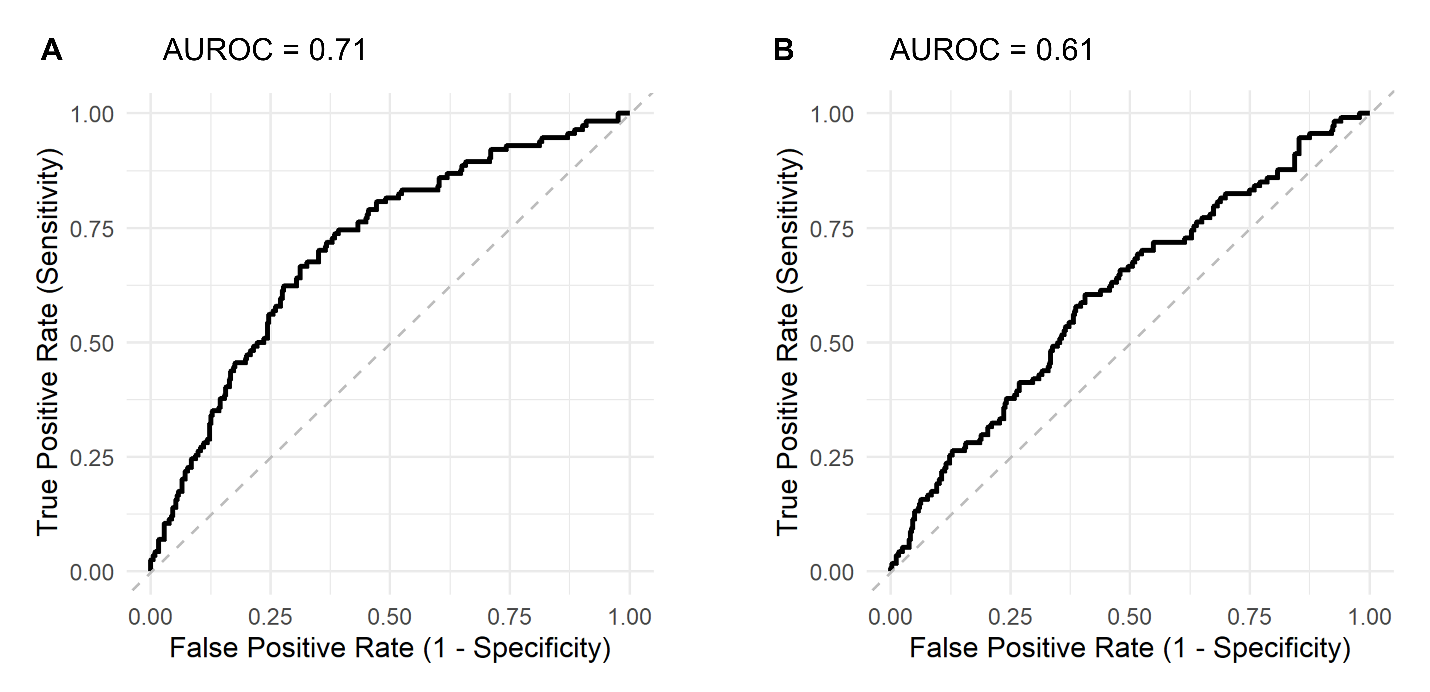


# Figure E5. Selection of soft-thresholding power (β) for WGCNA.

The soft-thresholding power (β) was selected to be 6 because A) it was the smallest power parameter that led to the network approximately fitting a scale-free topology with an *R^2^* close to 0.9 (*R^2^* = 0.89, red line), and B) the number of connections in the network did not significantly decrease when the power parameter reached 6.


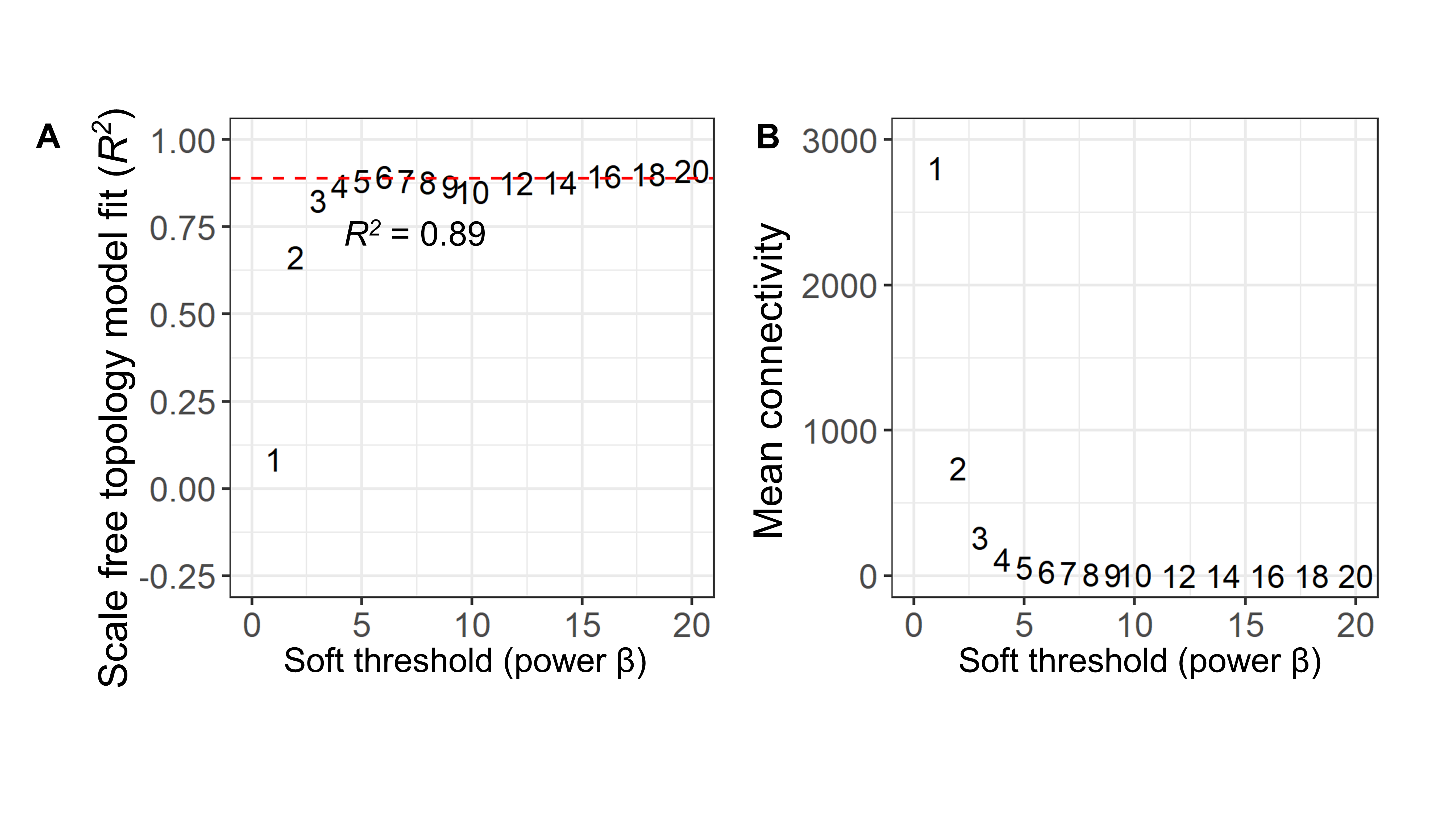


References

1. Reilly JP, Anderson BJ, Hudock KM, Dunn TG, Kazi A, Tommasini A, Charles D, Shashaty MG, Mikkelsen ME, Christie JD, et al. Neutropenic sepsis is associated with distinct clinical and biological characteristics: A cohort study of severe sepsis. *Crit Care* 2016;20(1):222.

2. Kan M, Shumyatcher M, Diwadkar A, Soliman G, Himes BE. Integration of transcriptomic data identifies global and cell-specific asthma-related gene expression signatures. *AMIA Annu Symp Proc* 2018;2018:1338-1347.

3. Kauffmann A, Gentleman R, Huber W. Arrayqualitymetrics--a bioconductor package for quality assessment of microarray data. *Bioinformatics* 2009;25(3):415-416.

4. Carvalho BS, Irizarry RA. A framework for oligonucleotide microarray preprocessing. *Bioinformatics* 2010;26(19):2363-2367.

5. Ritchie ME, Phipson B, Wu D, Hu Y, Law CW, Shi W, Smyth GK. Limma powers differential expression analyses for rna-sequencing and microarray studies. *Nucleic Acids Res* 2015;43(7):e47.

6. Scicluna BP, van Vught LA, Zwinderman AH, Wiewel MA, Davenport EE, Burnham KL, Nürnberg P, Schultz MJ, Horn J, Cremer OL, et al. Classification of patients with sepsis according to blood genomic endotype: A prospective cohort study. *The Lancet Respiratory Medicine* 2017;5(10):816-826.

7. Ota M, Nagafuchi Y, Hatano H, Ishigaki K, Terao C, Takeshima Y, Yanaoka H, Kobayashi S, Okubo M, Shirai H, et al. Dynamic landscape of immune cell-specific gene regulation in immune-mediated diseases. *Cell* 2021;184(11):3006-3021 e3017.

8. Ward Jr JH. Hierarchical grouping to optimize an objective function. *Journal of the American statistical association* 1963;58(301):236-244 %@ 0162-1459.

9. Hosack DA, Dennis G, Jr., Sherman BT, Lane HC, Lempicki RA. Identifying biological themes within lists of genes with ease. *Genome Biol* 2003;4(10):R70.

10. Huang da W, Sherman BT, Lempicki RA. Systematic and integrative analysis of large gene lists using david bioinformatics resources. *Nat Protoc* 2009;4(1):44-57.

11. Korotkevich G, Sukhov V, Budin N, Shpak B, Artyomov MN, Sergushichev A. Fast gene set enrichment analysis.2016.

12. Ritchie ME, Phipson B, Wu D, Hu Y, Law CW, Shi W, Smyth GK. Limma powers differential expression analyses for rna-sequencing and microarray studies. *Nucleic Acids Research* 2015;43(7):e47-e47.

13. Langfelder P, Zhang B, Horvath S. Defining clusters from a hierarchical cluster tree: The dynamic tree cut package for r. *Bioinformatics* 2008;24(5):719-720.

14. Zhang B, Horvath S. A general framework for weighted gene co-expression network analysis. *Stat Appl Genet Mol Biol* 2005;4:Article17.

15. Ronner L, Giannini HM, Miano TA, Ittner CAG, Turner AP, Dunn TG, Agyekum RS, Dasgupta A, West K, Jones TK, et al. Parsimonious subphenotyping algorithms perform differently in patients with sepsis and hematologic malignancy. *Crit Care Med* 2025.
